# *In vitro* and *in silico* insights into tyrosinase inhibitors with (*E*)-benzylidene-1-indanone derivatives

**DOI:** 10.1016/j.csbj.2019.07.017

**Published:** 2019-08-01

**Authors:** Hee Jin Jung, Sang Gyun Noh, Yujin Park, Dongwan Kang, Pusoon Chun, Hae Young Chung, Hyung Ryong Moon

**Affiliations:** a College of Pharmacy, Pusan National University, Busan 46241, Republic of Korea; b Longevity Life Science and Technology Institutes, Pusan National University, Busan 46241, Republic of Korea; c Aging Tissue Bank, College of Pharmacy, Pusan National University, Busan 46241, Republic of Korea; d College of Pharmacy, Inje University, Gimhae 47392, Republic of Korea

**Keywords:** 1-Indanone, 2,4-Dihydroxy benzylidene, Melanin, Tyrosinase inhibitor

## Abstract

Tyrosinase is a key enzyme responsible for melanin biosynthesis and is effective in protecting skin damage caused by ultraviolet radiation. As part of ongoing efforts to discover potent tyrosinase inhibitors, we systematically designed and synthesized thirteen (*E*)-benzylidene-1-indanone derivatives (**BID1**–**13**) and determined their inhibitory activities against tyrosinase. Among the compounds evaluated, **BID3** was the most potent inhibitor of mushroom tyrosinase (IC_50_ = 0.034 µM, monophenolase activity; IC_50_ = 1.39 µM, diphenolase activity). Kinetic studies revealed that **BID3** demonstrated a mixed type of tyrosinase inhibition with *K*_i_ value of 2.4 µM using l-DOPA as a substrate. *In silico* molecular docking simulations demonstrated that **BID3** can bind to the catalytic and allosteric sites of tyrosinase to inhibit enzyme activity which confirmed *in vitro* experimental studies between **BID3** and tyrosinase. Furthermore, melanin contents decreased and cellular tyrosinase activity was inhibited after **BID3** treatment. These observations revealed that **BID3** is a potent tyrosinase inhibitor and potentially could be used as a whitening agent for the treatment of pigmentation-related disorders.

## 1 Introduction

Melanin is synthesized by melanocytes in the vassal layer of the epidermis generally refers to the family of pigments commonly known for protecting mammalian skin from damage caused by harmful UV radiation by scavenging free radicals or dispersing incoming UV light [1]. However, abundant generation of melanin can cause visible hyperpigmentation of the epidermis, which may be obvious as melasma, freckles, age spots, or senile lentigines [2].

Tyrosinase (EC 1.14.18.1), a copper-containing metalloenzyme, is a key enzyme involved in melanogenic processes. Tyrosinase catalyzes the two rate-limiting steps in melanogenesis; hydroxylation of tyrosine (cresolase or monophenolase activity) to produce 3,4-dihydroxyphenylalanine (l-DOPA) and the subsequent oxidation of l-DOPA (catecholase or diphenolase activity) to the corresponding DOPA-quinone. When l-tyrosine is the substrate, the product of tyrosinase catalyzed reaction is DOPA-quinone, which is then converted to melanin [3]. These steps are crucial for the protection of skin against UV damage. Mushroom *Agaricus bisporus* is used to its commercially available and high homology with mammalian tyrosinase enzyme that renders it well suited as a model for studies on melanogenesis [4]. In humans, melanin helps defend skin form the damage caused by UV [5]. However, the excess level of melanin can cause various dermatological disorders including hyperpigmentations, melisma, freckles, and age spots [6]. Therefore, new tyrosinase inhibitors exhibiting drug-like and skin-whitening properties are required to inhibit excessive skin pigmentation.

1-Indanone, with a benzocyclopentanone skeleton, is considered as the rigid cousins of chalcones, incorporating the α,β-unsaturated ketone system of chalcones forming a cyclic 5 membered ring, which is a naturally occurring component present in various edible plant sources [7]. Several studies have shown that compounds possessing a 1-indanone moiety have pharmacological importance, as they exhibit various beneficial biological activities, including anti-inflammatory [8], [9], [10], anticancer [11], [12], antioxidant [13], anti-Parkinson’s disease [14], anti-Alzheimer disease [15], [16], [17], antimicrobial [18], anti-immune suppressive [19], and anti-tyrosinase [20] properties.

Chalcones (1,3-diaryl-2-propen-1-ones) are aromatic ketones consisting of two aromatic rings linked to a three carbon α,β-unsaturated carbonyl system as a central core [21], [22]. These are naturally occurring component found in various edible natural plants and considered as precursor compounds for synthetic route of flavonoids and isoflavonoids such as pyrazolines, pyrimidine, flavanol, flavones, flavanones, isollavone, aurones, antocianidin, dihydroflavanol and dihydrochalcone [23], [24], [25]. The α,β-unsaturated carbonyl system is paramount to the type of biological activity, since these system are distribute in many natural products like chalcones as well as indanone, where the molecular are relatively more planar, and the transmission of the electronic effects of the aryl substituents are directed to the carbonyl group [7]. Previous many researcher reported that chalcones were highlighted as a new class of tyrosinase inhibitor in several publication [26], [27], [28], [29]. Recently, Kim and co-worker [30], [31] designed and synthesized a series of chalcone derivatives and evaluated them for their *in vitro* anti-tyrosinse and anti-melanogenic activities against a murine melanocytes.

According to our previously reported data, derivatives with an (*E*)-β-phenyl-α,β-unsaturated carbonyl scaffold showed potent tyrosinase inhibition [32], [33], [34]. (*E*)-Benzylidene-1-indanones can be an attractive anti-melanogenic agent, since the compounds have the (*E*)-β-phenyl-α,β-unsaturated carbonyl scaffold in their chemical structure. Notably, Radhakrishnan et al. (2015) [20] designed and synthesized a series of benzylidene-1-indanone derivatives bearing a (*Z*)-configuration and evaluated them for anti-tyrosinase activity. Although these (*Z*)-benzylidene-1-indanone derivatives have been investigated anti-tyrosinase and anti-melanogenic activities of (*E*)-benzylidene-1-indanone derivatives have yet to be scrutinized.

Therefore, we designed and synthesized thirteen compounds (**BID1**–**13**) of the 1-indanone skeleton bearing a (*E*)-form benzylidene. Among these synthetic compounds, with 2,4-dihydroxy group on the B ring of 1-indanone showed the greatest tyrosinase inhibition (approximately 400-fold more effective than kojic acid, positive control). Moreover, we further evaluate **BID3** tyrosinase inhibitory activity as well as characterize its regulatory role in melanin synthesis using B16F10 melanoma cells. In extended experiments, we evaluated anti-melanogenic potential of **BID3**, and sought to identify the enzyme kinetics and molecular docking studies. In successive experiments **BID3** cellular tyrosinase potential and melanin production in α-MSH and IBMX-induced B16F10 melanoma cells was also explored.

## 2 Materials and methods

### 2.1 Reagents

Mushroom tyrosinase (EC 1.14.18.1), alpha-melanocyte stimulating hormone (α-MSH), 3-isobutyl-1-methylxanthine (IBMX), l-tyrosine, l-3,4-dihydroxyphenylalanine (l-DOPA), dimethyl sulfoxide (DMSO), kojic acid, phthalic acid, and *trans*-cinnamic acid were purchased from Sigma-Aldrich (St. Louis, MO, USA). Dulbecco’s modified Eagle’s medium (DMEM), fetal bovine serum (FBS), streptomycin, and amphotericin were purchased from WELGENE Inc. (Gyeongsan-si, South Korea). All other reagents were purchased from Sigma-Aldrich.

### 2.2 Chemistry

#### 2.2.1 General methods

All reagents were obtained commercially and used without further purification. Thin layer chromatography (TLC) and column chromatography were conducted on Merck precoated 60F245 plates and MP Silica 40–63, 60 Å, respectively. High resolution (HR) mass spectroscopy data were obtained on an Agilent Accurate Mass quadruple-time of flight (Q-TOF) liquid chromatography (LC) mass spectrometer (Agilent, Santa Clara, CA, USA) in ESI positive mode. Nuclear magnetic resonance (NMR) spectra were recorded on a Varian Unity INOVA 400 spectrometer or a Varian Unity AS500 spectrometer (Agilent Technologies, Santa Clara, CA, USA) for ^1^H NMR (400 and 500 MHz) and for ^13^C NMR (100 MHz), respectively. DMSO‑*d*_6_, CD_3_OD, and CDCl_3_ were used as NMR solvents for NMR samples. Coupling constant (*J*) and chemical shift values were measured in hertz (Hz) and parts per million (ppm), respectively. The abbreviations used in the analysis of ^1^H NMR data are as follows: s (singlet), brs (broad singlet), d (doublet), dd (doublet of doublets), t (triplet), td (triplet of doublets), q (quartet), and m (multiplet).

#### 2.2.2 Procedure for the synthesis of **BID1**–**13**

A solution of benzaldehyde (1.1–1.2 equiv.) and 1-indanone (100 mg, 0.76 mmol) in 1 M in HCl acetic acid (0.4 mL) was stirred at room temperature for 4–48 h. After the addition of water, the precipitates were filtered and washed with water and hexane:ethyl acetate (1:1), hexane:dichloromethane (4:1–1:1), or a mixture of dichloromethane and methanol, depending on the properties of the remaining benzaldehydes to give (*E*)-benzylidene-1-indanones (**BID1**–**13**) in yields of 32.8–99.1%. Structural characterization (^1^H and ^13^C NMR and mass data of all BIDs) of synthesized compounds is provided in Supplementary information.

##### 2.2.2.1 (*E*)-2-(4-Hydroxybenzylidene)-2,3-dihydro-1*H*-inden-1-one (**BID1**)

Yellow solid; yield, 93.4%; reaction time, 6 h; molecular formula, C_16_H_12_O_2_; mp, 224.7–225.9 °C; ^1^H NMR (400 MHz, DMSO‑*d*_6_) *δ* 10.10 (s, 1H, OH), 7.72 (d, 1H, *J* = 7.6 Hz, 7-H), 7.64–7.58 (m, 4H, 4-H, 5-H, 2′-H, 6′-H), 7.43 (s, 1H, vinylic H), 7.39 (t, 1H, *J* = 7.2 Hz, 6-H), 6.87 (d, 2H, *J* = 8.0 Hz, 3′-H, 5′-H), 3.99 (s, 2H, CH_2_); ^13^C NMR (100 MHz, DMSO‑*d*_6_) *δ* 193.9, 160.1, 150.4, 138.3, 135.1, 134.0, 133.6, 132.2, 128.2, 127.2, 126.7, 124.1, 116.7, 32.6; HRMS (ESI + ) *m*/*z* C_16_H_13_O_2_ (M+H)^+^ calcd 237.0910, obsd 237.0911.

##### 2.2.2.2 (*E*)-2-(3,4-Dihydroxybenzylidene)-2,3-dihydro-1*H*-inden-1-one (**BID2**)

Yellow solid; yield, 86.6%; reaction time, 5 h; molecular formula, C_16_H_12_O_3_; mp, 251.2–252.4 °C; ^1^H NMR (400 MHz, DMSO‑*d*_6_) *δ* 9.65 (s, 1H, OH), 9.24 (s, 1H, OH), 7.72 (d, 1H, *J* = 7.6 Hz, 7-H), 7.66–7.61 (m, 2H, 4-H, 5-H), 7.42 (t, 1H, *J* = 7.2 Hz, 6-H), 7.35 (s, 1H, vinylic H), 7.19 (s, 1H, 2′-H), 7.08 (d, 1H, *J* = 8.0 Hz, 6′-H), 6.83 (d, 1H, *J* = 8.0 Hz, 5′-H), 3.98 (s, 2H, CH_2_); ^13^C NMR (100 MHz, DMSO‑*d*_6_) *δ* 193.8, 150.4, 148.7, 146.3, 138.3, 135.1, 134.5, 132.0, 128.2, 127.3, 127.1, 125.0, 124.0, 118.2, 116.7, 32.7; HRMS (ESI+) *m*/*z* C_16_H_13_O_3_ (M+H)^+^ calcd 253.0859, obsd 253.0857.

##### 2.2.2.3 (*E*)-2-(2,4-Dihydroxybenzylidene)-2,3-dihydro-1*H*-inden-1-one (**BID3**)

Yellow solid; yield, 50.9%; reaction time, 10 h; molecular formula, C_16_H_12_O_3_; mp, 198.5–199.9 °C (dec.); ^1^H NMR (500 MHz, CD_3_OD) *δ* 8.17 (s, 1H, vinylic H), 7.82 (d, 1H, *J* = 8.0 Hz, 6′-H), 7.67–7.63 (m, 3H, 4-H, 5-H, 7-H), 7.45 (t, 1H, *J* = 7.0 Hz, 6-H), 6.45 (d, 1H, *J* = 8.5 Hz, 5′-H), 6.39 (s, 1H, 3′-H), 4.01 (s, 2H, CH_2_); ^13^C NMR (100 MHz, DMSO‑*d*_6_) *δ* 193.9, 161.6, 160.4, 150.3, 138.6, 134.8, 131.7, 130.3, 128.7, 128.1, 127.2, 123.9, 114.3, 108.6, 103.1, 32.7; HRMS (ESI+) *m*/*z* C_16_H_13_O_3_ (M+H)^+^ calcd 253.0859, obsd 253.0858.

##### 2.2.2.4 (*E*)-2-(4-Hydroxy-3-methoxybenzylidene)-2,3-dihydro-1*H*-inden-1-one (**BID4**)

Yellow solid; yield, 52.0%; reaction time, 4 h; molecular formula, C_17_H_14_O_3_; mp, 186.9–187.4 °C; ^1^H NMR (400 MHz, DMSO‑*d*_6_) *δ* 9.71 (s, 1H, OH), 7.73 (d, 1H, *J* = 7.6 Hz, 7-H), 7.67–7.62 (m, 2H, 4-H, 5-H), 7.45 (d, 1H, *J* = 2.0 Hz, 2′-H), 7.43 (t, 1H, *J* = 7.2 Hz, 6-H), 7.31 (s, 1H, vinylic H), 7.24 (dd, 1H, *J* = 8.0, 2.0 Hz, 6′-H), 6.87 (d, 1H, *J* = 8.0 Hz, 5′-H), 4.05 (s, 2H, CH_2_), 3.84 (s, 3H, OCH_3_); ^13^C NMR (100 MHz, DMSO‑*d*_6_) *δ* 193.9, 150.5, 149.7, 148.5, 138.3, 135.2, 134.4, 132.3, 128.2, 127.3, 127.1, 125.7, 124.1, 116.6, 115.4, 56.3, 32.5; HRMS (ESI + ) *m*/*z* C_17_H_15_O_3_ (M+H)^+^ calcd 267.1016, obsd 267.1016.

##### 2.2.2.5 (*E*)-2-(3-Ethoxy-4-hydroxybenzylidene)-2,3-dihydro-1*H*-inden-1-one (**BID5**)

Yellow solid; yield, 60.1%; reaction time, 2 d; molecular formula, C_18_H_16_O_3_; mp, 135.8–136.4 °C; ^1^H NMR (500 MHz, CDCl3) *δ* 7.90 (d, 1H, *J* = 7.5 Hz, 7-H), 7.61 (s, 1H, vinylic H), 7.60 (td, 1H, *J* = 7.5, 1.0 Hz, 5-H), 7.55 (d, 1H, *J* = 7.5 Hz, 4-H), 7.42 (td, 1H, *J* = 7.5, 1.0 Hz, 6-H), 7.28 (dd, 1H, *J* = 8.0, 2.0 Hz, 6′-H), 7.14 (d, 1H, *J* = 2.0 Hz, 2′-H), 7.01 (d, 1H, *J* = 8.0 Hz, 5′-H), 6.05 (s, 1H, OH), 4.20 (q, 2H, *J* = 7.0 Hz, CH_2_CH_3_), 4.00 (s, 2H, CH_2_), 1.50 (t, 3H, *J* = 7.0 Hz, CH_2_CH_3_); ^13^C NMR (100 MHz, CDCl_3_) *δ* 194.2, 149.6, 147.8, 146.2, 138.5, 134.6, 134.6, 132.5, 128.2, 127.8, 126.3, 125.0, 124.5, 115.2, 114.4, 64.9, 32.6, 15.0; HRMS (ESI+) *m*/*z* C_18_H_17_O_3_ (M+H)^+^ calcd 281.1172, obsd 281.1172.

##### 2.2.2.6 (*E*)-2-(3-Hydroxy-4-methoxybenzylidene)-2,3-dihydro-1*H*-inden-1-one (**BID6**)

Yellow solid; yield, 85.9%; reaction time, 4 h; molecular formula, C_17_H_14_O_3_; mp, 186.1–186.9 °C; ^1^H NMR (400 MHz, DMSO‑*d*_6_) *δ* 9.27 (s, 1H, OH), 7.72 (d, 1H, *J* = 7.2 Hz, 7-H), 7.65–7.59 (m, 2H, 4-H, 5-H), 7.41 (t, 1H, *J* = 7.2 Hz, 6-H), 7.37 (s, 1H, vinylic H), 7.21 (s, 1H, 2′-H), 7.18 (d, 1H, *J* = 8.4 Hz, 6′-H), 6.98 (d, 1H, *J* = 8.4 Hz, 5′-H), 3.98 (s, 2H, CH_2_), 3.79 (s, 3H, CH_3_); ^13^C NMR (100 MHz, DMSO‑*d*_6_) *δ* 193.9, 150.4, 150.3, 147.4, 138.2, 135.2, 134.0, 133.0, 128.4, 128.3, 127.3, 124.6, 124.1, 117.6, 112.8, 56.3, 32.6; HRMS (ESI+) *m*/*z* C_17_H_15_O_3_ (M+H)^+^ calcd 267.1016, obsd 267.1009.

##### 2.2.2.7 (*E*)-2-(4-Methoxybenzylidene)-2,3-dihydro-1*H*-inden-1-one (**BID7**)

White solid; yield, 32.8%; reaction time, 6 h; molecular formula, C_17_H_14_O_2_; mp, 138.9–139.5 °C; ^1^H NMR (400 MHz, CDCl_3_) *δ* 7.88 (d, 1H, *J* = 7.6 Hz, 7-H), 7.63 (s, 1H, vinylic H), 7.63–7.56 (m, 3H, 5-H, 2′-H, 6′-H), 7.53 (d, 1H, *J* = 8.0 Hz, 4-H), 7.40 (t, 1H, *J* = 7.2 Hz, 6-H), 6.96 (d, 2H, *J* = 8.4 Hz, 3′-H, 5′-H), 3.98 (s, 2H, CH_2_), 3.84 (s, 3H, OCH_3_); ^13^C NMR (100 MHz, CDCl_3_) *δ* 194.6, 161.1, 149.7, 138.5, 134.5, 134.0, 132.8, 132.6, 128.4, 127.8, 126.3, 124.5, 114.7, 55.6, 32.7; HRMS (ESI+) *m*/*z* C_17_H_15_O_2_ (M+H)^+^ calcd 251.1067, obsd 251.1064.

##### 2.2.2.8 (*E*)-2-(3,4-Dimethoxybenzylidene)-2,3-dihydro-1*H*-inden-1-one (**BID8)**

Yellowish solid; yield, 80.5%; reaction time, 2 d; molecular formula, C_18_H_16_O_3_; mp, 176.5–177.2 °C; ^1^H NMR (500 MHz, CDCl_3_) *δ* 7.90 (d, 1H, *J* = 7.5 Hz, 7-H), 7.62 (s, 1H, vinylic H), 7.60 (td, 1H, *J* = 7.0, 1.0 Hz, 5-H), 7.55 (d, 1H, *J* = 7.5 Hz, 4-H), 7.42 (t, 1H, *J* = 7.5 Hz, 6-H), 7.30 (dd, 1H, *J* = 8.0, 2.0 Hz, 6′-H), 7.18 (d, 1H, *J* = 2.0 Hz, 2′-H), 6.95 (d, 1H, *J* = 8.0 Hz, 5′-H), 4.01 (s, 2H, CH_2_), 3.96 (s, 3H, OCH_3_), 3.94 (s, 3H, OCH_3_); ^13^C NMR (100 MHz, CDCl_3_) *δ* 194.5, 150.8, 149.6, 149.3, 138.4, 134.6, 134.3, 132.8, 128.6, 127.8, 126.3, 124.8, 124.5, 113.7, 111.5, 56.2, 56.2, 32.6.

##### 2.2.2.9 (*E*)-2-(2,4-Dimethoxybenzylidene)-2,3-dihydro-1*H*-inden-1-one (**BID9**)

Yellow solid; yield, 99.1%; reaction time, 24 h; molecular formula, C_18_H_16_O_3_; mp, 74.0.-74.6 °C; ^1^H NMR (500 MHz, CDCl_3_) *δ* 8.12 (s, 1H, vinylic H), 7.91 (d, 1H, *J* = 7.5 Hz, 7-H), 7.66 (d, 1H, *J* = 8.5 Hz, 6′-H), 7.58 (t, 1H, *J* = 7.5 Hz, 5-H), 7.53 (d, 1H, *J* = 7.5 Hz, 4-H), 7.41 (t, 1H, *J* = 7.5 Hz, 6-H), 6.58 (dd, 1H, *J* = 8.5, 2.5 Hz, 5′-H), 6.49 (d, 1H, *J* = 2.5 Hz, 3′-H), 3.97 (s, 2H, CH_2_), 3.89 (s, 3H, OCH_3_), 3.87 (s, 3H, OCH3); ^13^C NMR (100 MHz, CDCl_3_) *δ* 194.6, 162.7, 161.0, 149.8, 138.8, 134.3, 132.4, 131.0, 128.6, 127.7, 126.2, 124.5, 117.9, 105.4, 98.5, 55.8, 55.7, 32.8; HRMS (ESI+) *m*/*z* C_18_H_17_O_3_ (M+H)^+^ calcd 281.1172, obsd 281.1180.

##### 2.2.2.10 (*E*)-2-(3,4,5-Trimethoxybenzylidene)-2,3-dihydro-1*H*-inden-1-one (**BID10**)

Beige solid; yield, 63.2%; reaction time, 6 h; molecular formula, C_19_H_18_O_4_; mp, 164.4–165.2 °C; ^1^H NMR (400 MHz, CDCl_3_) *δ* 7.87 (d, 1H, *J* = 7.2 Hz, 7-H), 7.59–7.52 (m, 3H, 4-H, 5-H, vinylic H), 7.39 (t, 1H, *J* = 7.2 Hz, 6-H), 6.86 (s, 2H, 2′-H, 6′-H), 3.99 (s, 2H, CH_2_), 3.91 (s, 6H, 2 × OCH_3_), 3.89 (s, 3H, OCH_3_); ^13^C NMR (100 MHz, CDCl_3_) *δ* 194.4, 153.6, 149.5, 140.0, 138.3, 134.8, 134.3, 134.0, 131.1, 127.9, 126.3, 124.6, 108.3, 61.2, 56.5, 32.4; HRMS (ESI+) *m*/*z* C_19_H_18_O_4_ (M+H)^+^ calcd 311.1278, obsd 311.1282.

##### 2.2.2.11 (*E*)-2-(4-Hydroxy-3,5-dimethoxybenzylidene)-2,3-dihydro-1*H*-inden-1-one (**BID11**)

Yellow solid; yield, 84.0%; reaction time, 5 h; molecular formula, C_18_H_16_O_4_; mp, 190.2–191.1 °C; ^1^H NMR (500 MHz, DMSO‑*d*_6_) *δ* 9.12 (s, 1H, OH), 7.76 (d, 1H, *J* = 7.5 Hz, 7-H), 7.69–7.66 (m, 2H, 4-H, 5-H), 7.49 (s, 1H, vinylic H), 7.47–7.44 (m, 1H, 6-H), 7.07 (s, 2H, 2′-H, 6′-H), 4.12 (s, 2H, CH_2_), 3.86 (s, 6H, 2 × OCH_3_); ^13^C NMR (100 MHz, DMSO‑*d*_6_) *δ* 193.9, 150.5, 148.7, 138.9, 138.2, 135.2, 134.8, 132.6, 128.2, 127.3, 125.9, 124.1, 109.5, 56.8, 32.4; HRMS (ESI+) *m*/*z* C_18_H_17_O_4_ (M+H)^+^ calcd 297.1121, obsd 297.1128.

##### 2.2.2.12 (*E*)-2-(3-Bromo-4-hydroxybenzylidene)-2,3-dihydro-1*H*-inden-1-one (**BID12**)

Yellowish beige solid; yield, 54.4%; reaction time, 10 h; molecular formula, C_16_H_11_BrO_2_; mp, 225.0–226.2 °C; ^1^H NMR (400 MHz, DMSO‑*d*_6_) *δ* 10.92 (s, 1H, OH), 7.89 (s, 1H, 2′-H), 7.72 (d, 1H, *J* = 7.6 Hz, 7-H), 7.67–7.64 (m, 2H, 4-H, 5-H), 7.61 (d, 1H, *J* = 8.4 Hz, 6′-H), 7.42 (t, 1H, *J* = 7.2 Hz, 6-H), 7.40 (s, 1H, vinylic H), 7.03 (d, 1H, *J* = 8.4 Hz, 5′-H), 4.02 (s, 2H, CH_2_); ^13^C NMR (100 MHz, DMSO‑*d*_6_) *δ* 193.8, 156.4, 150.5, 138.0, 136.0, 135.4, 133.6, 132.4, 132.4, 128.4, 128.3, 127.3, 124.1, 117.3, 110.7, 32.4; HRMS (ESI+) *m*/*z* C_16_H_12_BrO_2_ (M+H)^+^ calcd 315.0015, obsd 315.0008, *m*/*z* C_16_H_12_BrO_2_ (M+2+H)^+^ calcd 316.9996, obsd 316.9996.

##### 2.2.2.13 (*E*)-2-(3,5-Dibromo-4-hydroxybenzylidene)-2,3-dihydro-1*H*-inden-1-one (**BID13**)

White solid; yield, 63.7%; reaction time, 24 h; molecular formula, C_16_H_10_Br_2_O_2_; mp, 267.4–268.1 °C; ^1^H NMR (400 MHz, DMSO‑*d*_6_) *δ* 10.55 (brs, 1H, OH), 7.92 (s, 2H, 2′-H, 6′-H), 7.73 (d, 1H, *J* = 7.2 Hz, 7-H), 7.67–7.63 (m, 2H, 4-H, 5-H), 7.43 (t, 1H, *J* = 7.2 Hz, 6-H), 7.38 (s, 1H, vinylic H), 4.05 (s, 2H, CH_2_); ^13^C NMR (100 MHz, DMSO‑*d*_6_) *δ* 193.8, 152.7, 150.7, 137.8, 135.6, 135.4, 135.1, 130.9, 130.2, 128.4, 127.4, 124.2, 112.8, 32.2; HRMS (ESI+) *m*/*z* C_16_H_11_Br_2_O_2_ (M+H)^+^ calcd 392.9120, obsd 392.9124, C_16_H_11_Br_2_O_2_ (M+2+H)^+^ calcd 394.9101, obsd 394.9101, C_16_H_11_Br_2_O_2_ (M+H)^+^ calcd 396.9082, obsd 396.9087.

### 2.3 Biological evaluation

#### 2.3.1 Mushroom tyrosinase inhibitory assay

Tyrosinase inhibitory activity assay was performed using mushroom tyrosinase as described previously, with minor modification [35], [36]. Briefly, 10 µL of a specified concentrations of each compound (0.0005–50 µM) and 20 µL of mushroom tyrosinase (1000 units/mL) in a 50 mM phosphate buffer (pH 6.5) were added to 170 µL reaction mixture in a 96-well microplate (Corning, USA). The ratio of 1 mM l-tyrosine or l-DOPA solution, 50 mM potassium phosphate buffer (pH 6.5), and distilled water was 10:10:9. The reaction mixtures were incubated at 37 °C for 30 min. After that, the amount of dopachrome produced in each well was measured by spectrophotometric analysis at 492 nm (OD_492_) using a microplate reader. The tyrosinase inhibition rate (%) was calculated as (1 − Abs_sample_/Abs_control_) × 100%. The degree of sample inhibition was expressed as the concentration required for 50% inhibition (IC_50_).

#### 2.3.2 Enzyme kinetic analysis with tyrosinase

To determine the kinetic mechanisms of inhibition, two complimentary kinetics methods were used: Lineweaver–Burk and Dixon plots [37], [38], [39]. Using Lineweaver-Burk plots (double reciprocal plots), the inhibition type of **BID3** was determined using various concentrations of l-DOPA (0.125, 0.25, 0.5, and 1 mM), as substrates, in the absence and presence of various concentrations of **BID3** (1, 2, and 4 μM). A Dixon plot (single reciprocal plots) for tyrosinase inhibition was obtained in the presence of various concentrations of l-DOPA (0.125, 0.25, 0.5, and 1 mM). The concentrations of **BID3** were 1, 2, and 4 µM. The enzymatic procedures consisted of the previously described tyrosinase assay methods. The inhibition constant (*K*_i_) was determined from the interpretation of Dixon plots.

#### 2.3.3 Tyrosinase molecular docking simulations

To determine the structure of the enzyme-inhibitor complex and to ensure accuracy, repeatability, and reliability of the docking results, we employed AutoDock4.2 software. For dicking studies, the crystal structures of mushroom tyrosinase protein target was obtained from the protein sequence alignment (Protein Data Bank (PDB ID: 2Y9X) (http://www.rcsb.org/adb) [40]. Automated docking simulations were performed between tyrosinase and kojic acid, phthalic acid, cinnamic acid, or **BID3**. For the docking procedure: conversed 2D into 3D structures, calculated charges, and added hydrogen atoms using the ChemOffice program (http://www.cambridgesoft.com) [41]. LigandScout 4.1.5 was used for the prediction of possible interactions between ligands and tyrosinase and the identification of pharmacophores.

#### 2.3.4 Cell culture and cell viability assay

Murine melanoma B16F10 cells were purchased from the Korean Cell Line Bank (KCLB, Seoul, Korea) and cultured in DMEM supplemented with 10% FBS and 1% streptomycin, and then incubated at 37 °C, humidified with 5% atmospheric CO_2_. Cell viability analyses were performed as previously described [42]. Briefly, cells seeded at a density of 1 × 10^4^ cells/well in a 96-well plate for 24 h. On the following day, the cells were exposed to different concentrations of **BID3** and incubated for 24 or 48 h, respectively. Then, 10 µL EZ-Cytox solution was added to each well and the cells were incubated for 2–4 h. Absorbance was determined using ELISA at a wavelength of 450 nm. Each assay was performed in triplicate.

#### 2.3.5 Melanin contents assay

Melanin content was determined as described previously [43]. B16F10 melanoma cells (2 × 10^5^ cells/well) were seeded in 6-well culture plates. To determine the inhibitory effect of **BID3** on melanogenesis, fresh medium was replaced with medium containing **BID3** (1, 5 and 10 μM) or kojic acid (10 µM) as a positive control for 1hr, and then stimulated with α-MSH (5 μM) and IBMX (200 µM) for 48 h. After washed twice with PBS, the cells were detached by incubation in Trypsin/EDTA, and the pellets were dissolved in 100 µL of 1 N NaOH, and then incubated at 60 °C for 1 h and mixed to solubilize the melanin. The melanin contents were determined by measuring absorbance at 405 nm by using the ELISA reader (TECAN, Sunrise, Austria). The melanin content was calculated using the following equation: (Δ_sample_/Δ_control_) × 100%. All determinations were performed in triplicate.

#### 2.3.6 Cellular tyrosinase activity

Cellular tyrosinase activity was assessed as described previously, with slight modifications [44], [45]. Briefly, 2 × 10^5^/cells were plated in 6-well dishes and incubated overnight. The cells were exposed to various concentrations of **BID3** (1, 5, and 10 µM) or kojic acid (10 µM) for 1 h, and subsequently stimulated with α-MSH (5 µM) and IBMX (200 µM) for 48 h. After being rinsed twice with PBS, the cells were lysed with 100 µL lysis solution containing 50 mM phosphate buffer (pH 6.5), 0.1 mM phenylmethylsulfonylfluoride (PMSF), and 1% Triton X-100 and frozen at −80 °C for 30 min. Lysates were thawed and centrifuged at 12,000 rpm for 30 min at 4 °C. Then, supernatant (80 µL) were combined with and 2 mg/mL l-DOPA (20 µL) in a 96-well plate. After incubation at 37 °C for 30 min, the optical density at 492 nm was measured using an ELISA reader (TECAN, Salzburg, Austria). The protein concentration was determined using BCA protein assay reagent using BCA as a standard (Thermo Scientific, Rockford, IL, USA).

#### 2.3.7 Statistical analysis

All data are presented as the mean ± standard error of the mean (S.E.M). The data were analyzed by one-way analysis of variance (ANOVA) to determined differences between treatments, followed by the Bonferroni post-hoc test. A value of *p* < 0.05 was considered statistically significant.

## 3 Results and discussion

### 3.1 Chemistry

**Scheme 1:**
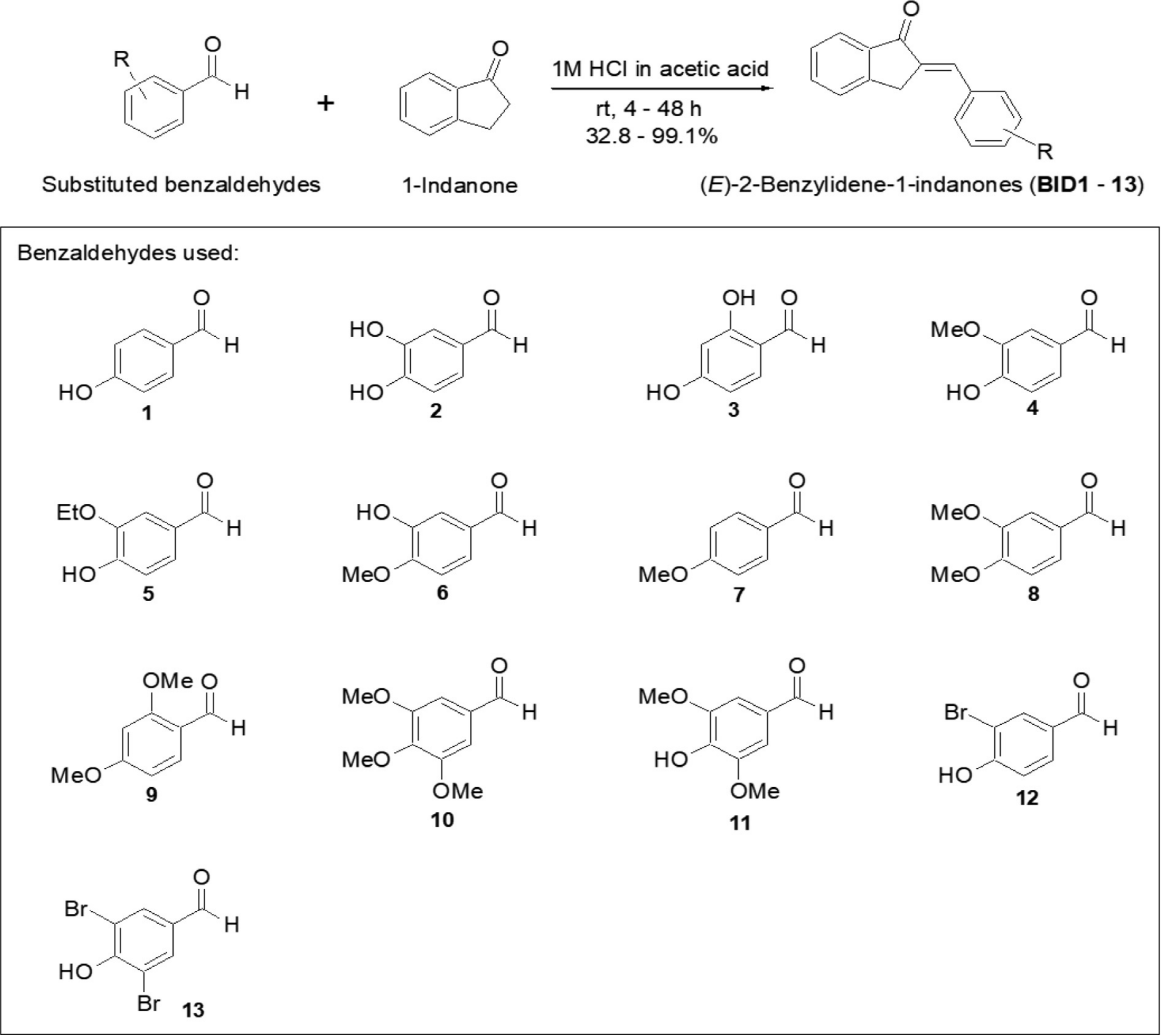
Synthesis of (*E*)-2-benzylidene-1-indanones.

The (*E*)-benzylidene-1-indanones derivatives (**BID1**–**13**) were synthesized according to the general Scheme 1. In this present study, thirteen (*E*)-2-benzylidene-1-indanone derivatives were synthesized and their tyrosinase inhibitory properties were examined. Target (*E*)-2-benzylidene-1-indanone derivatives were synthesized employing acidic (1 M HCl in acetic acid) conditions. These reactions generated the target (*E*)-2-benzylidene-1-indanone derivatives in yields of 32.8–99.1%. It appears that only the *E*-isomers of 2-benzylidene-1-indanones were generated due to the A-strain known as the 1,3-allylic strain. The A-strain (steric hindrance) between the phenyl ring and the carbonyl group in the *Z*-isomer is apparently larger than that between the phenyl ring and the methylene group in the *E*-isomer. The structures of synthetic compounds were verified by ^1^H and ^13^C NMR and mass spectrometry as described in the experimental section (Figs. S1–S38). In particular, the chemical shift of olefinic proton is deshielded in the *E*-isomer of 2-benzylidene due to the anisotropic effect of the carbonyl group and appears downfield (above 7 ppm) compared to the Z isomer (<7 ppm) [46]. The chemical shifts of olefinic protons in the benzylidene-1-indanones appeared in the range of 7.31–8.17 ppm. Therefore, the benzylidene-1-indanone derivatives was determined to be (*E*)-stereoisomers. We found that the presence of hydroxyl or methoxyl group at the 2-position of the phenyl ring moves the chemical shift of the olefinic proton to 0.5–0.8 ppm downfield.

### 3.2 Tyrosinase inhibitory properties of compounds

The tyrosinase inhibitory potential of the (*E*)-benzylidene-1-indanones derivatives were examined using mushroom tyrosinase. The monophenolase (l-tyrosine) and diphenolase (l-DOPA) were used as substrates for these experiments [35]. Enzyme reactions were performed with tyrosinase, substrate, and test inhibitors. All tested compounds demonstrated a concentration-dependent inhibition. The IC_50_ values of the (*E*)-benzylidene-1-indanones derivatives are shown in Table 1.

**Table 1:** Mushroom tyrosinase inhibitory potential of the substituted 2,3-dihydro-1*H*-inden-1-one (1-indanone) chalcone-like derivatives (**BID1**–**13**).

**Figure:**
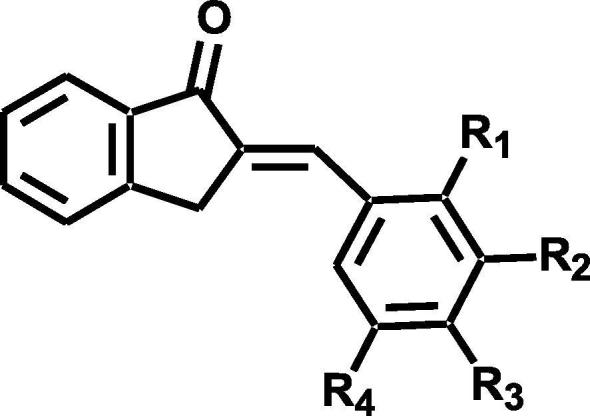

Compounds	R_1_	R_2_	R_3_	R_4_	IC_50_ (μM)^a^	IC_50_ (μM)^b^
BID1	H	H	OH	H	39.74 ± 3.71	130.0 ± 5.39
BID2	H	OH	OH	H	41.27 ± 3.03	52.93 ± 2.62
BID3	OH	H	OH	H	0.034 ± 0.00224	1.39 ± 0.00004
BID4	H	OMe	OH	H	73.51 ± 5.78	>200
BID5	H	OEt	OH	H	>200	>200
BID6	H	OH	OMe	H	5.00 ± 0.91	50.78 ± 2.16
BID7	H	H	OMe	H	>200	>200
BID8	H	OMe	OMe	H	>200	>200
BID9	OMe	H	OMe	H	>200	96.85 ± 9.78
BID10	H	OMe	OMe	OMe	>200	>200
BID11	H	OMe	OH	OMe	75.15 ± 2.24	>200
BID12	H	Br	OH	H	>200	>200
BID13	H	Br	OH	Br	>200	75.31 ± 7.94
Kojic acid^c^					13.77 ± 0.20	33.14 ± 0.93
Phthalic acid^d^					>200	>200
Cinnamic acid^d^					>200	>200

The IC_50_ values (µM) were calculated from a log dose inhibition curve using l-tyrosine^a^ and l-DOPA^b^ as a substrate, respectively and are as means ± SEM of triplicate experiments. ^c^Reported competitive type inhibitor. ^d^Reported mixed type inhibitor.

**Fig. 1:**
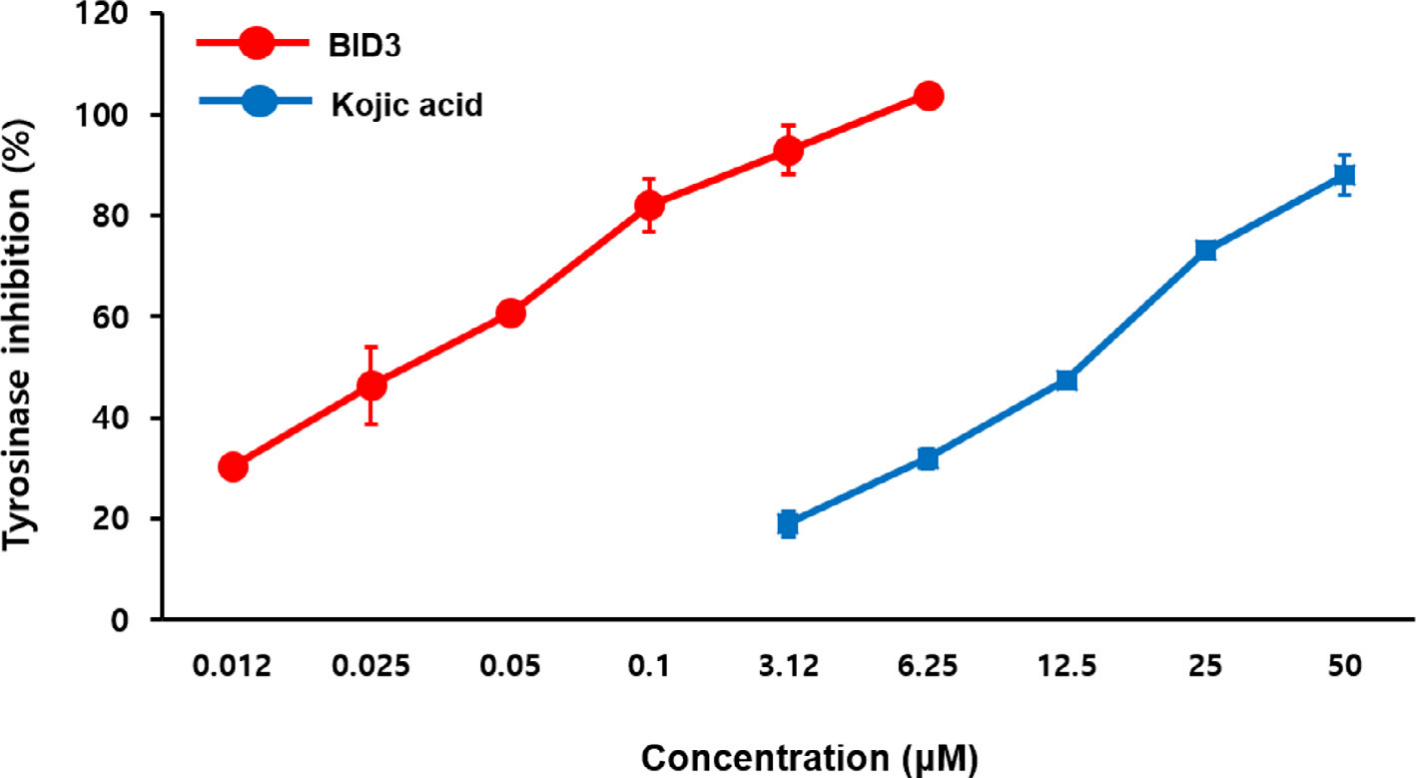
Concentration-dependent inhibition of mushroom tyrosinase activity by **BID3** and kojic acid (positive control) (*n* = 3).

In both l-tyrosine and l-DOPA substrates, **BID3** exhibited strong inhibitory activitt with IC_50_ values of 0.034 ± 0.00224 µM and 1.39 ± 0.00004 µM, respectively, compared with the positive control kojic acid, which had IC_50_ values of 13.77 ± 0.20 µM and 33.14 ± 0.93 µM, respectively (Fig. 1A). Furthermore, **BID6** showed potent activity toward l-tyrosine and l-DOPA with IC_50_ values of 5.00 ± 0.91 µM and 53.11 ± 2.79 µM, respectively. **BID1** showed significant inhibition against tyrosinase via l-tyrosine and l-DOPA pathways, with IC_50_ values of 39.74 ± 3.71 µM and 130.0 ± 5.39 µM, respectively. **BID4** and **BID11** displayed moderate tyrosinase inhibitory activity. Other derivatives were inactive. Likewise, **BID2** showed significant activity toward l-tyrosine and l-DOPA, with IC_50_ values of 41.27 ± 3.03 µM and 52.93 ± 2.62 µM. In addition, reported mixed type inhibitors, phthalic acid and cinnamic acid showed no inhibitory effect at the concentration of 200 µM [47].

Consistent with the findings of structure-activity relationship (SAR) studies, derivatives containing (*E*)-benzylidene-1-indanone substituents on the phenyl ring noticeably influenced the potential tyrosinase inhibition. **BID3**, which inhibited tyrosinase with the highest potency, bear a 2-hydroxy group in the presence of a 4-hydroxy group and greatly inhibited tyrosinase activity (**BID1** *vs* **BID3**). These results suggested that the 2,4-dihydroxy group (resorcinol moiety) in the phenyl ring structure may be responsible for the enhanced inhibition of tyrosinase. Interestingly, in a previous study on potential synthetic tyrosinase inhibitors, we showed that the 2,4-dihydroxy substitution potently inhibited tyrosinase activity [48], [49]. In addition, our SAR data also indicated that the introduction of a 3-hydroxy group in the presence of a 4-hydroxy group affects tyrosinase inhibition. These phenomena were demonstrated by the finding that a 4-methoxy-3-hydroxy phenyl ring increased inhibitory activity against tyrosinase, whereas the introduction of a 3,4-dihydroxy group (catechol moiety) decreased tyrosinase inhibitory activity (**BID2** *vs* **BID6**) [35]. There results suggest that the 3-hydroxy-4-methoxy group is also responsible for tyrosinase inhibitory activity. Nevertheless, in our present study, **BID9** and **BID11**, which contain 2,4-dimethoxyphenyl or 3,5-dimethoxyphenyl groups, showed moderate tyrosinase inhibitory activity. Based on IC_50_ values via the l-tyrosine and l-DOPA pathway, **BID3** demonstrated the most potent tyrosinase inhibitory activity therefore, we further studied the mechanism underlying this inhibitory effect. Radhakrishnan et al. (2015) [20] reported that hydroxyl substituted (*Z*)-benzylidene-1-indanone compounds potent tyrosinase inhibitors. Although previous studies have shown that hydroxyl-substituted (*Z*)-benzylidene-1-indanone derivatives possess anti-tyrosinase activity, to the best of our knowledge, there have been no reports of tyrosinase inhibition by (*E*)-benzylidene-1-indanone derivatives using cell-based experiments.

### 3.3 Enzyme kinetic analysis of tyrosinase inhibition

**Fig. 2:**
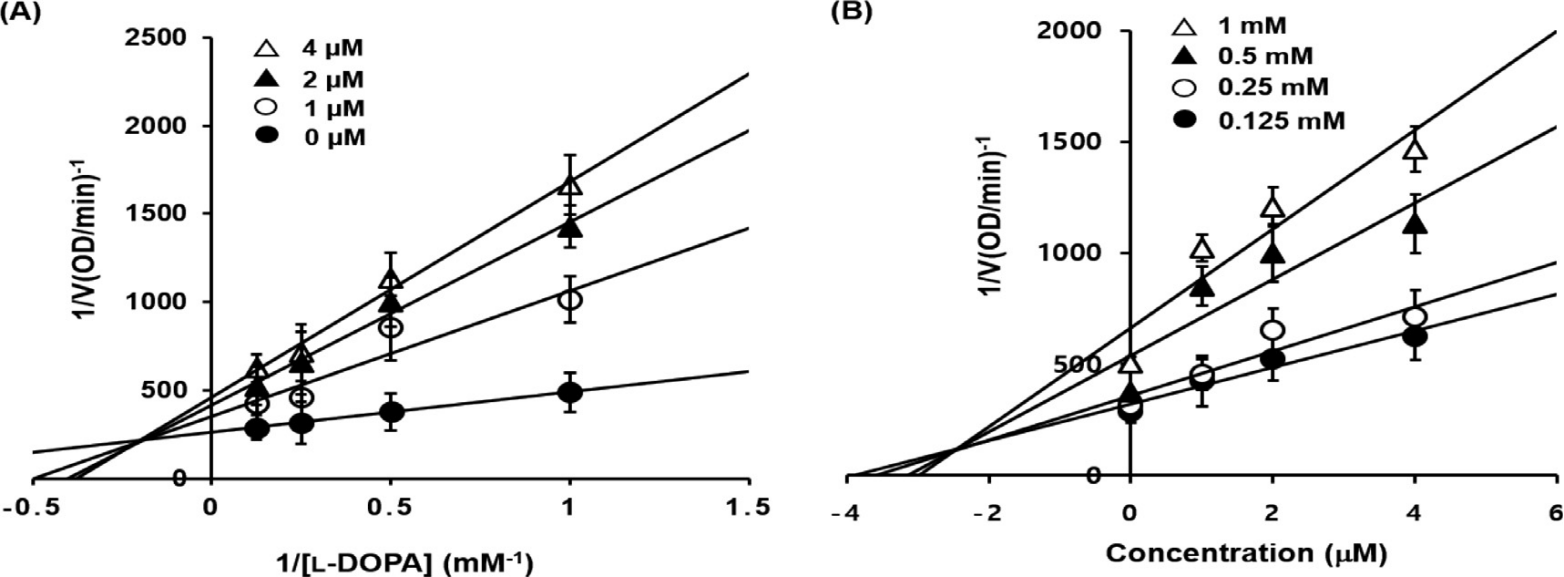
Lineweaver-Burk (A) and Dixon plots (B) for the inhibition of mushroom tyrosinase by **BID3** using l-DOPA as the substrate. The effects are shown in the presence of different concentrations of **BID3** [0 µM (*closed circle*), 1 µM (*open circle*), 2 µM (*closed triangle*), and 4 µM (*open triangle*)] and in the presence of different concentrations of substrate [0.125 mM (*closed circle*), 0.25 mM (*open circle*), 0.5 mM (*closed triangle*), and 1 mM (*open triangle*)] of l-DOPA. Error bars indicate the standard error of the mean (SEM).

Since **BID3** was the most potent inhibitor, we further studied the mechanism underlying its inhibitory effect. In an attempt to explain the mode of enzyme inhibition, kinetic analyses were performed at various concentrations of l-DOPA and various **BID3** concentrations. Dixon and Lineweaver-Burk plots were drawn using the data obtained from the kinetic studies in order to confirm the inhibition pattern and the inhibition constants (*K*_i_) were determined by interpretation of Dixon plots (Fig. 2A and B). The concentration of l-DOPA is denoted as follows: 1, 2, 4 mM. The intersection lies on the left side, indicating mixed-type inhibition against tyrosinase with a *K*_i_ value 2.4 µM (Fig. 2B). The *K*_i_ value represent the concentrations required to form an enzyme inhibitor complex, so inhibitor with lower *K*_i_ value indicate greater tyrosinase inhibition activity.

**Fig. 3:**
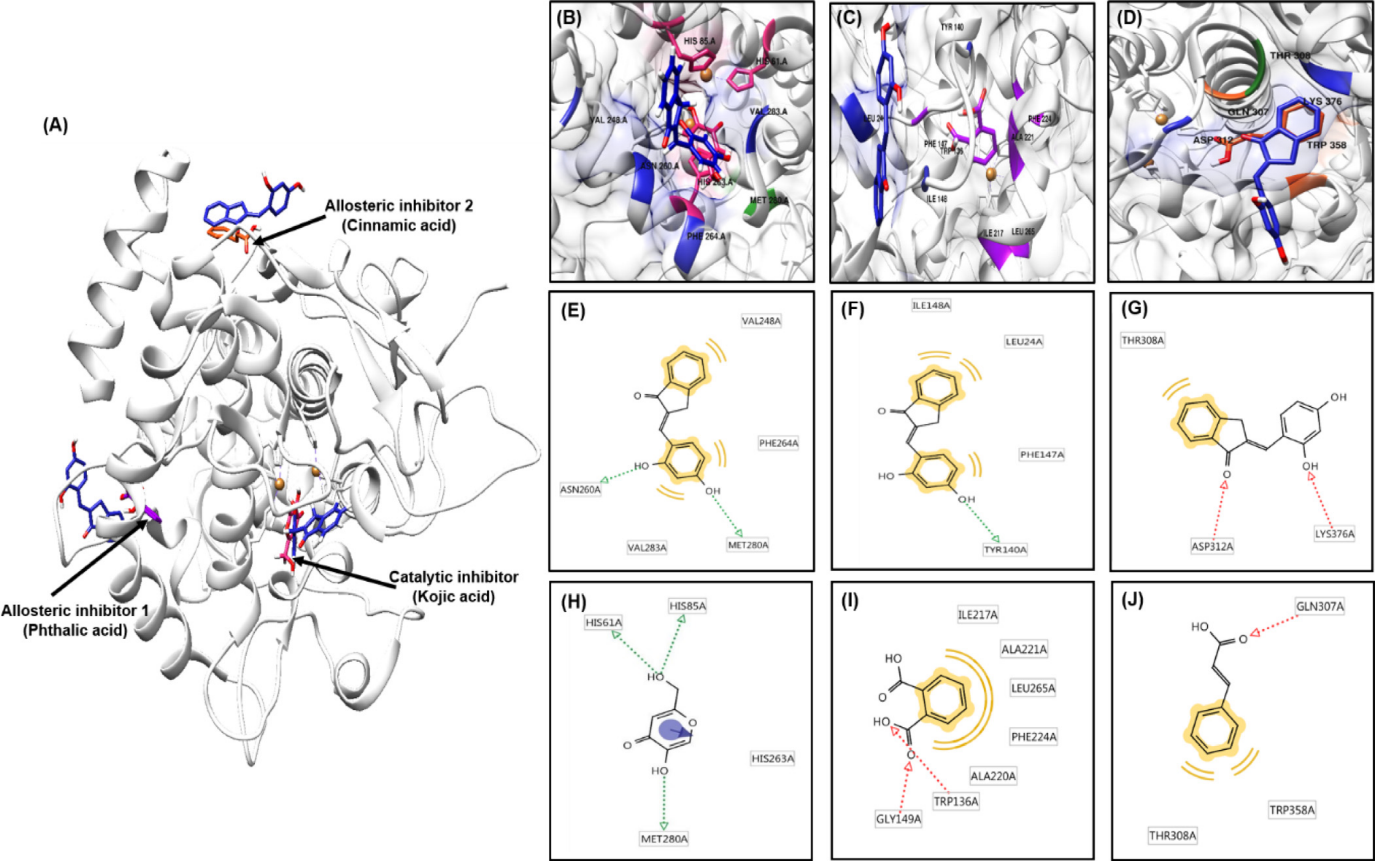
Molecular docking models of tyrosinase inhibition by kojic acid, phthalic acid, cinnamic acid, and **BID3** (blue color) (A). Inhibition mode of **BID3** at the tyrosinase catalytic site with the catalytic inhibitor, kojic acid (pink color) (B) and at allosteric sites 1 and 2 with the allosteric inhibitor, phthalic acid (violet color) (C) and cinnamic acid (orange color) (D), respectively. 2D ligand interaction diagrams of the catalytic (E) and allosteric (F and G) of tyrosinase by **BID3** and kojic acid (H), phthalic acid (I), and cinnamic acid (J) as catalytic and allosteric inhibitors, respectively. Green and red arrow: hydrogen-bonding, yellow: hydrophobic interaction. (For interpretation of the references to color in this figure legend, the reader is referred to the web version of this article.)

Molecular docking has contributed important insights into drug discovery over many years. However, docking procedures aim to identify the correct positions of ligands in the binding pocket of a protein and to predict the affinity between ligand and protein. In other words, docking describes a process through which two molecules fit together in a three-dimensional space. To determine whether **BID3** binds to the active site of tyrosinase, molecular docking analysis was performed to identify the possible binding positions of **BID3** in the crystal structure of mushroom tyrosinase (PDB ID: 2Y9X) (Fig. 3A). These results were calculated using the AutoDock4.2. program. The most stable binding position was that with the lowest score [50]. Recently, Hassani et al. [47] reported that cinnamic acid and phthalic acid were mixed-mode tyrosinase inhibitor. Thus, kojic aid, cinnamic acid and phthalic acid were used to validate docking results [51].

The molecular docking models of **BID3** and kojic acid (well-known competitive type inhibitor) in the catalytic site of tyrosinase are illustrated in Fig. 3B [52]. The tyrosinase-**BID3** inhibitor complex presented −6.28 kcal/mol binding energy including two hydrogen bonds with the ASN260 and MET280 residue of tyrosinase, and hydrophobic interactions were observed between **BID3** and tyrosinase residues VAL248, PHE264, and VAL283, which further stabilized the interaction in the catalytic site for mushroom tyrosinase (Table 2, and Fig. 3E and H). Moreover, molecular docking models of **BID3**, and of phthalic acid (allosteric inhibitor 1) and cinnamic acid (allosteric inhibitor 2) in the two allosteric sites are illustrated in Fig. 3C and 3D. Phthalic acid and cinnamic acid were taken as reference ligands at allosteric sites 1 and 2, respectively [47], [51]. As shown in Fig. 3F and I, **BID3** and phthalic acid exhibited one hydrogen bond with the TRY140 residue of tyrosinase, and three hydrophobic interactions residues with the LEU24, PHE147, and ILE148 of tyrosinase at allosteric site 1. The corresponding ligand interactions of **BID3** and cinnamic acid in the allosteric site 2 of tyrosinase included hydrogen bonds between ASP312 and LYS376 of tyrosinase, whereas THR308 interacted hydrophobically at allosteric site 2 (Fig. 3G and J). Furthermore, **BID3**-tyrosinase binding was found to exert binding energy in both allosteric sites of tyrosinase (−4.38 and −5.68 kcal/mol, respectively), indicating high binding affinity with both allosteric sites. Based on enzyme kinetic studies, **BID3** exerted a mixed-type inhibition, binding ability of **BID3** with both the catalytic site and two allosteric sites confirmed its mixed-type inhibition of tyrosinase.

**Table 2:** Binding sites and docking scores of **BID3** in mushroom tyrosinase (PDB ID: 2Y9X) as determined using AutoDock4.2 program.

	Compounds	Binding energy (kcal/mol)^a^	Binding residues^b^
Catalytic inhibitor	**BID3**	–6.28	VAL248, ASN260, PHE264, MET280, VAL283
	Kojic acid^c^	–5.23	HIS61, HIS85, HIS263, MET280

Allosteric inhibitor1	**BID3**	–4.38	LEU24, TRY140, PHE147, ILE148
	Phthalic acid^d^	–3.19	TRP136, GLY149, ILE217, ALA220, ALA221, PHE224, LEU265

Allosteric inhibitor2	**BID3**	–5.68	THR308, ASP312, LYS376
	Cinnamic acid^d^	–4.08	GLN307, THR308, TRP358

a Binding energy indicate binding affinity and capacity for the active site of tyrosinase enzyme.

b All amino acid residues from the enzyme–inhibitor complex were determined using AutoDock4.2 program.

c Reported competitive type inhibitor.

d Reported mixed type inhibitors.

### 3.4 Biological activity

**Fig. 4:**
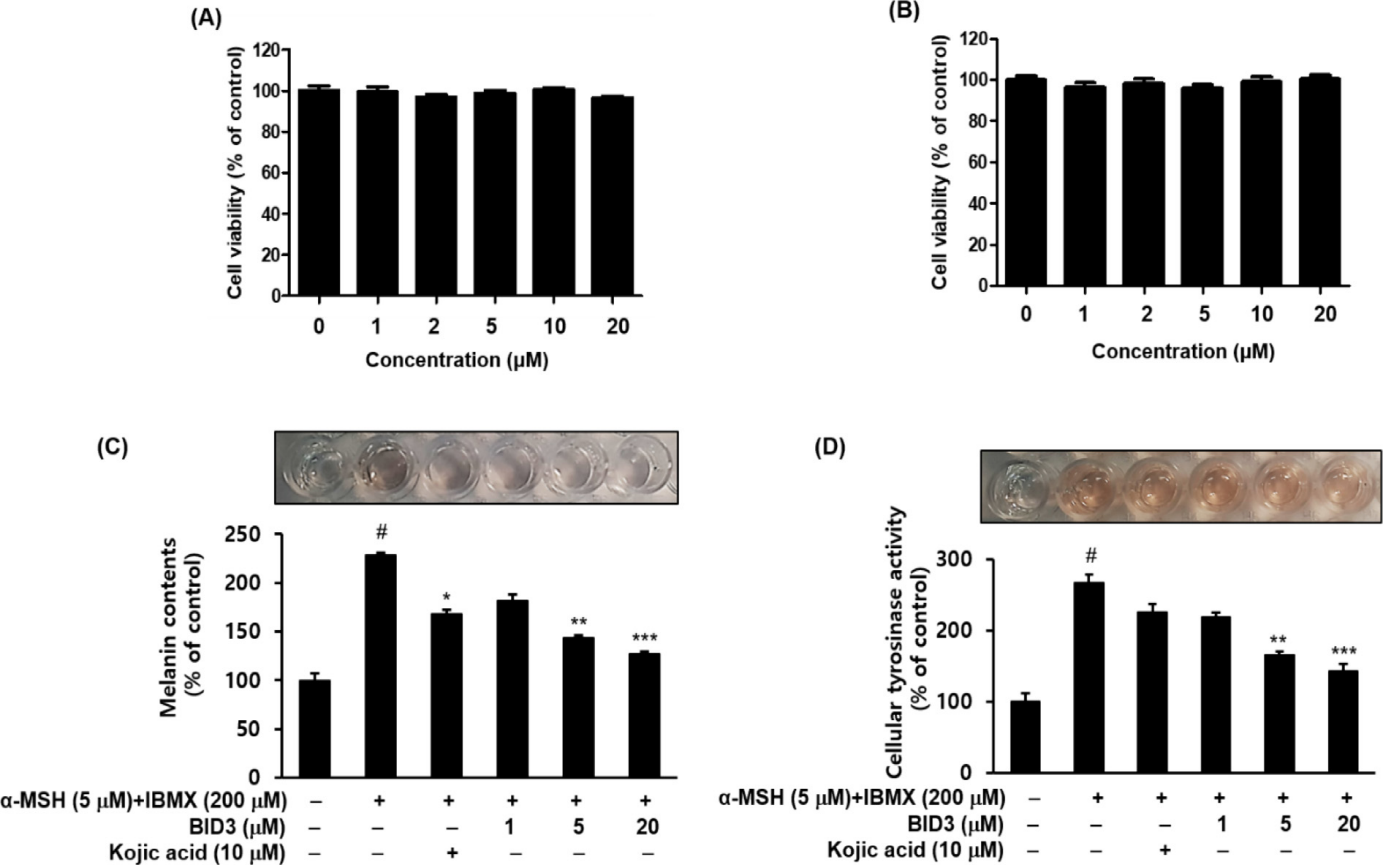
Effects of **BID3** on melanogenesis in B16F10 melanoma cells. Effect of **BID3** on B16F10 cell viability. Viability of cells treated with **BID3** (1–20 µM) for 24 h (A) and 48 h (B). Cell viability was determined using EZ-Cytox solution. The melanin contents (C) and intracellular tyrosinase activity (D) of B16F10 cells were determined after incubation with **BID3** or kojic acid (10 µM) for 48 h. Each experiment was conducted in triplicate, and the data represent the as mean ± SEM. ^#^*P* < 0.05 compared with the control; *p < 0.05, **p < 0.01 and ***p < 0.001, compared with the α-MSH and IBMX-treated.

To exploit the anti-tyrosinase effects of **BID3** in the cell culture model, we examined its cytotoxic effects on B16F10 cells. Cells were treated with different concentration of **BID3** (1–20 µM) for 48 h and assessed using the EZ-Cytox assay. The results indicated that **BID3** had no significant cytotoxic effect in B16F10 melanoma cells up to 20 µM (Fig. 4A and B). Therefore, subsequent experiments were performed using **BID3** up to 20 µM.

Melanogenesis is regulated by a tyrosinase enzymatic cascade. For this reason, several skin-whitening compounds have been developed to decrease tyrosinase activity. Kojic acid, a tyrosinase inhibitor, was used as positive control [53]. Thus, to investigate the effects of **BID3** on cAMP elevation induced hyperpigmentation, B16F10 cells were treated with α-MSH and IBMX in the presence of **BID3** (1, 5, and 20 µM) for 48 h, and melanin contents, and cellular tyrosinase activities were determined (Fig. 4C and D). Treatment with α-MSH and IBMX induced a significant increase (*P* < 0.001^#^) in melanin contents (Fig. 4C) and, cellular tyrosinase activity (Fig. 4D) in B16F10 cells compared with the untreated control. However, **BID3** treatment significantly (*P* < 0.01**; *P* < 0.001***) and concentration dependently decrease melanin contents and cellular tyrosinase activity of B16F10 cells compared with α-MSH or IBMX treated cells, indicating that the decrease in cellular melanin might be due to the inhibition of tyrosinase activity. Thus, these findings clearly shown that **BID3** exerted anti-melanogenic effects inhibiting tyrosinase activity and melanin synthesis in B16F10 cells without inducing cytotoxicity.

Indanone is one of the privileged structures of medicinal chemistry and is generally associated with variety of pharmacologically active compounds [54]. The indanone moiety is present in variety of physiologically active natural products. In view of this, Okpekon et al., [55] reported a novel 1-indanone compound, afzeliindanone, is isolated from *Uvaria afzelii* roots. This compound is the first 1-indanone derivative isolated from plants. Nagle et al., [56] reported a new methylated indane-aldehyde was isolated from marine cyanobacterium as tumor angiogenesis regulator. Likewise, since the biological importance of indanone core, past several years researchers have been focused on developing of pharmacologically active indanone analogs as therapeutic agents. The structural diversity of 1-indanones implies variety of biological responses and these compounds may be applied in agriculture and medicine. Various indanone based compounds have been developed as anti-Alzheimer’s disease [16], [57], [58], [59], anticancer [60], [61], [62], antimicrobial [63], [64], and antiviral agents [65], [66]. Due to the wide application potential, 1-indanones were interesting objects for further research and it is desirable to design new classes for their synthesis.

## 4 Conclusion

To identify new potent tyrosinase inhibitors, we have designed and synthesized thirteen (*E*)-benzylidene-1-indanone derivatives. Among these compounds, (*E*)-2-(2,4-dihydroxybenzylidene)-2,3-dihydro-1*H*-inden-1-one (**BID3**) potently inhibited tyrosinase activity (IC_50_ = 0.034 and 1.39 µM, for l-tyrosine and l-DOPA, respectively), which was better than reference compound, kojic acid (IC_50_ = 13.77 µM and 33.14 µM, respectively). Structure activity relationship revealed that presence of the dihydroxyl group at the 2 and 4-position of the (*E*)-benzylidene-1-indanone skeleton improved the activity against tyrosinase. The mode of enzymatic inhibition of the enzyme, determined by Lineweaver-Burk and Dixon plots, indicated that **BID3** is a mixed-type inhibitor. Molecular docking studies demonstrates that binding at the active site and at allosteric sites are an important mechanisms towards tyrosinase inhibitory activity. Based on these results, **BID3** seemed to be a potent tyrosinase inhibitor for the treatment of skin disorders such as hyperpigmentation.

## Declaration of Competing Interest

The authors declare that they have no known competing financial interests or personal relationships that could have appeared to influence the work reported in this paper.
