# Supplementary material for: In vitro and in silico insights into tyrosinase inhibitors with (E)-benzylidene-1-indanone derivatives
Source: Comput Struct Biotechnol J. 2019 Aug 1;17:1255–64. doi: 10.1016/j.csbj.2019.07.017 (PMC6944710; doi:10.1016/j.csbj.2019.07.017)
Supplement: Supplementary data 1 [file mmc1.docx]

**Supporting Information**

**For**

***In vitro* and *in silico* insights into tyrosinase inhibitors with (*E*)-benzylidene-1-indanone derivatives**

Hee Jin Jung^1,2,3^, Sang Gyun Noh^1,2,3^, Yujin Park^1^, Dongwan Kang^1^, Pusoon Chun^4^, Hae Young Chung^1,2,3,*^, Hyung Ryong Moon^1,*^

*^1^College of Pharmacy, Pusan National University, Busan 46241, Republic of Korea. ^2^Longevity life Science and Technology Institutes, Pusan National University, Busan 46241, Republic of Korea. ^3^Aging Tissue Bank, College of Pharmacy, Pusan National University, Busan 46241, Republic of Korea. ^4^College of Pharmacy, Inje University, Gimhae, 47392, Republic of Korea.*


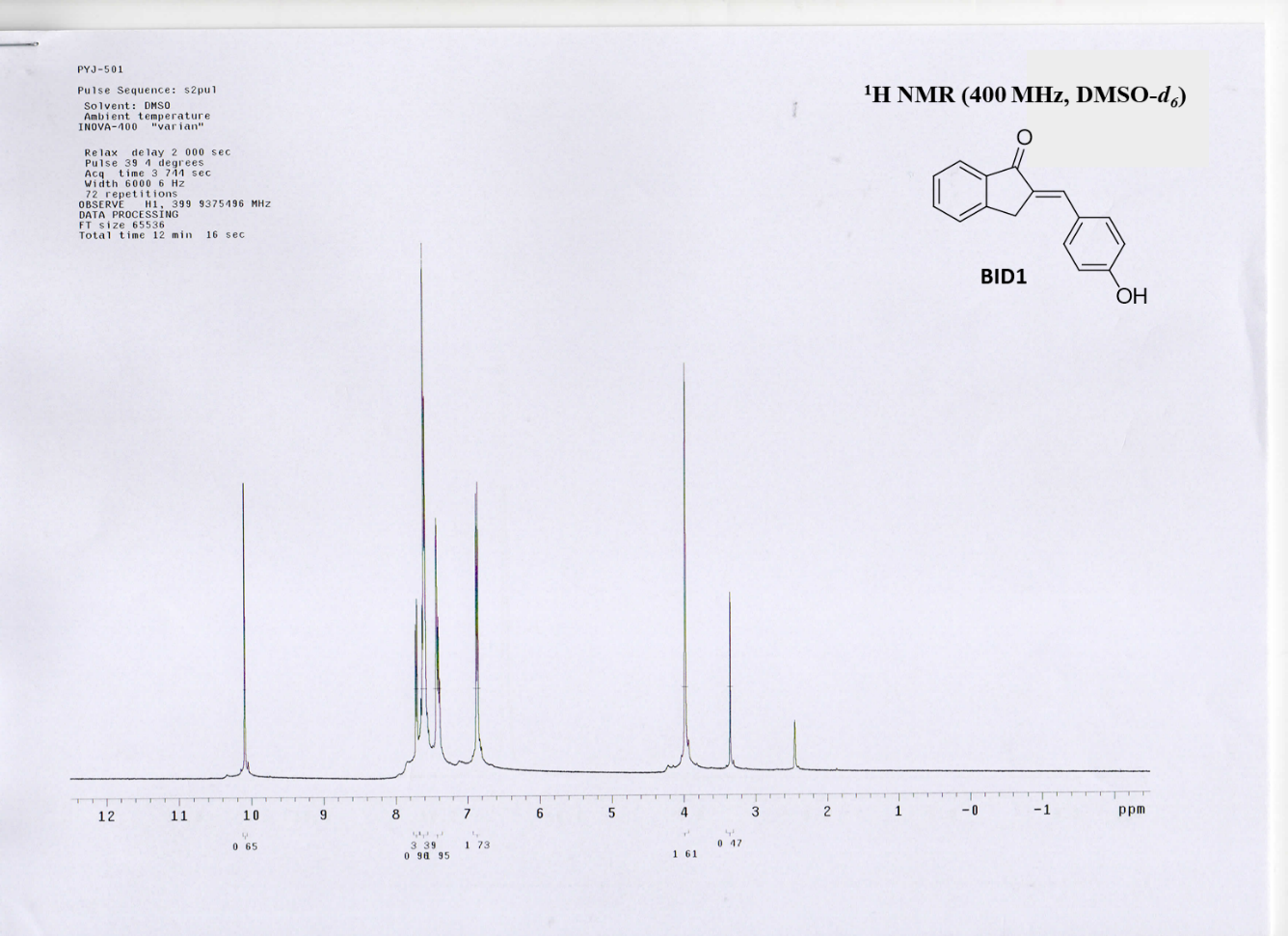


Fig. S1. ^1^H-NMR spectrum of **BID1**.


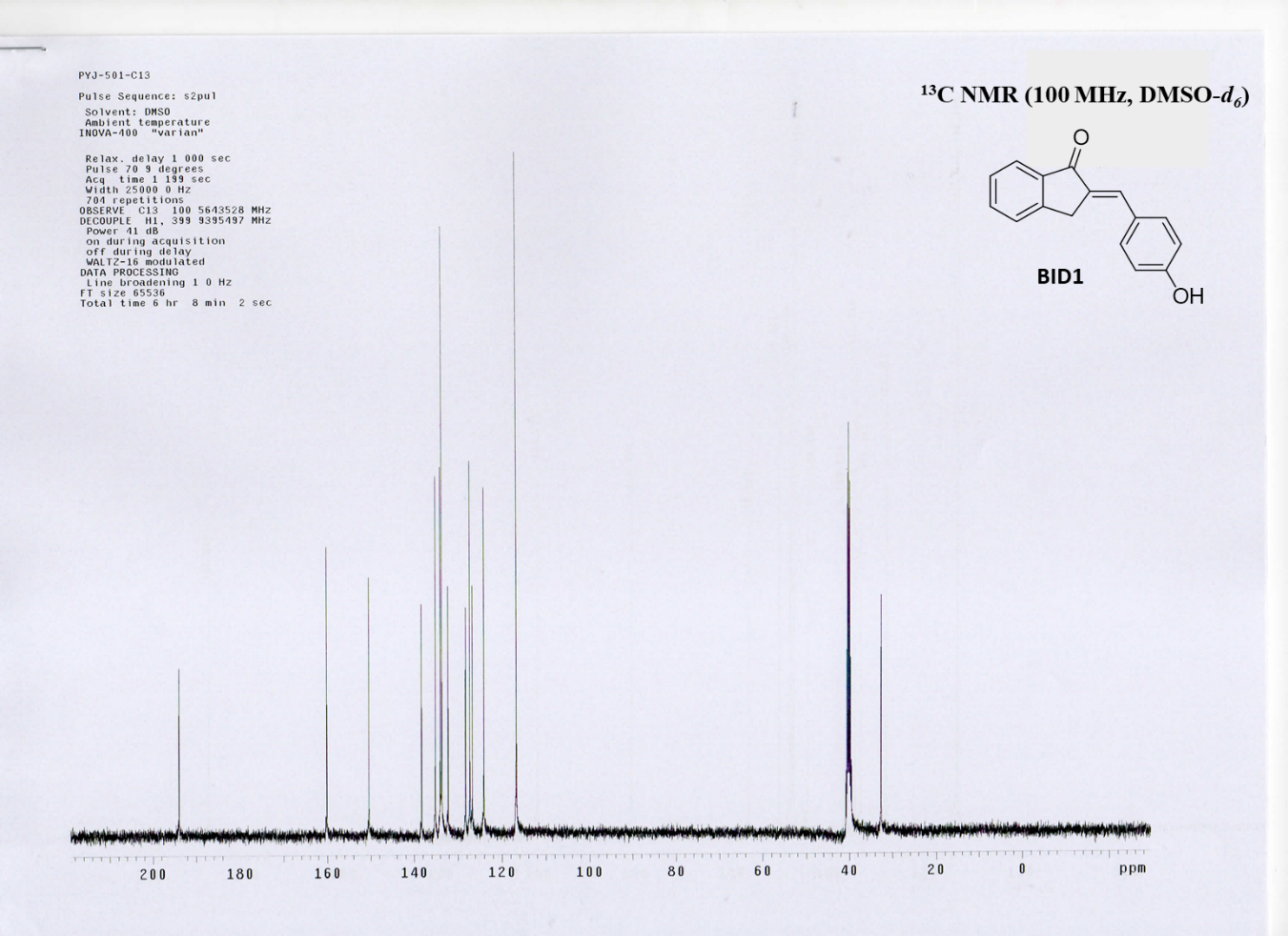


Fig. S2. ^13^C-NMR spectrum of **BID1**.


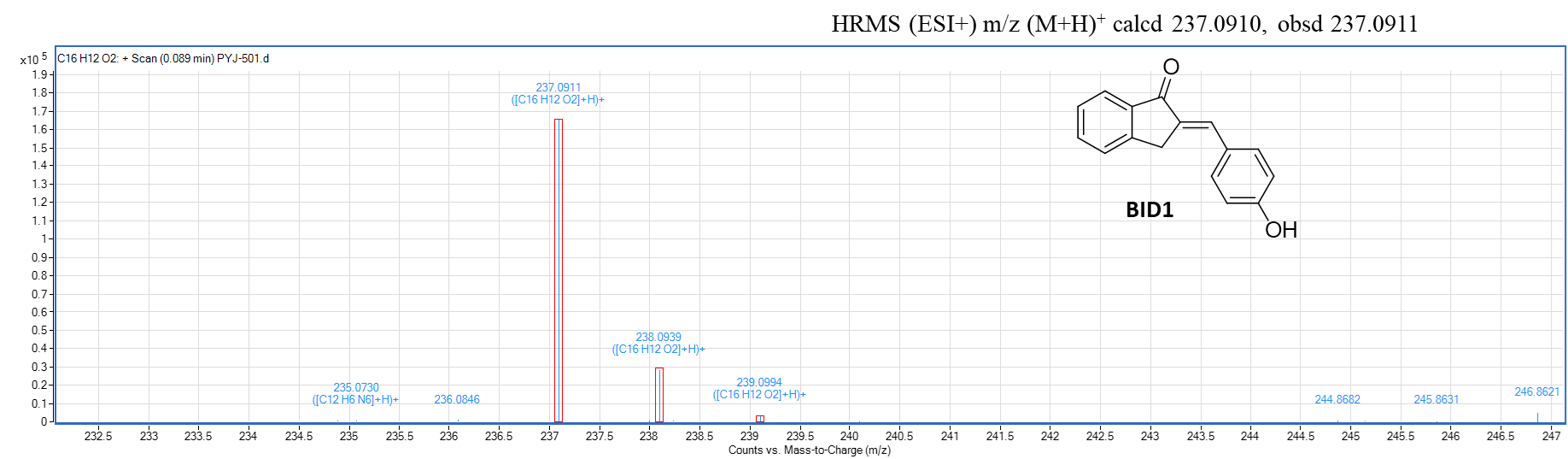


Fig. S3. ESI-MS spectrum of **BID1**.


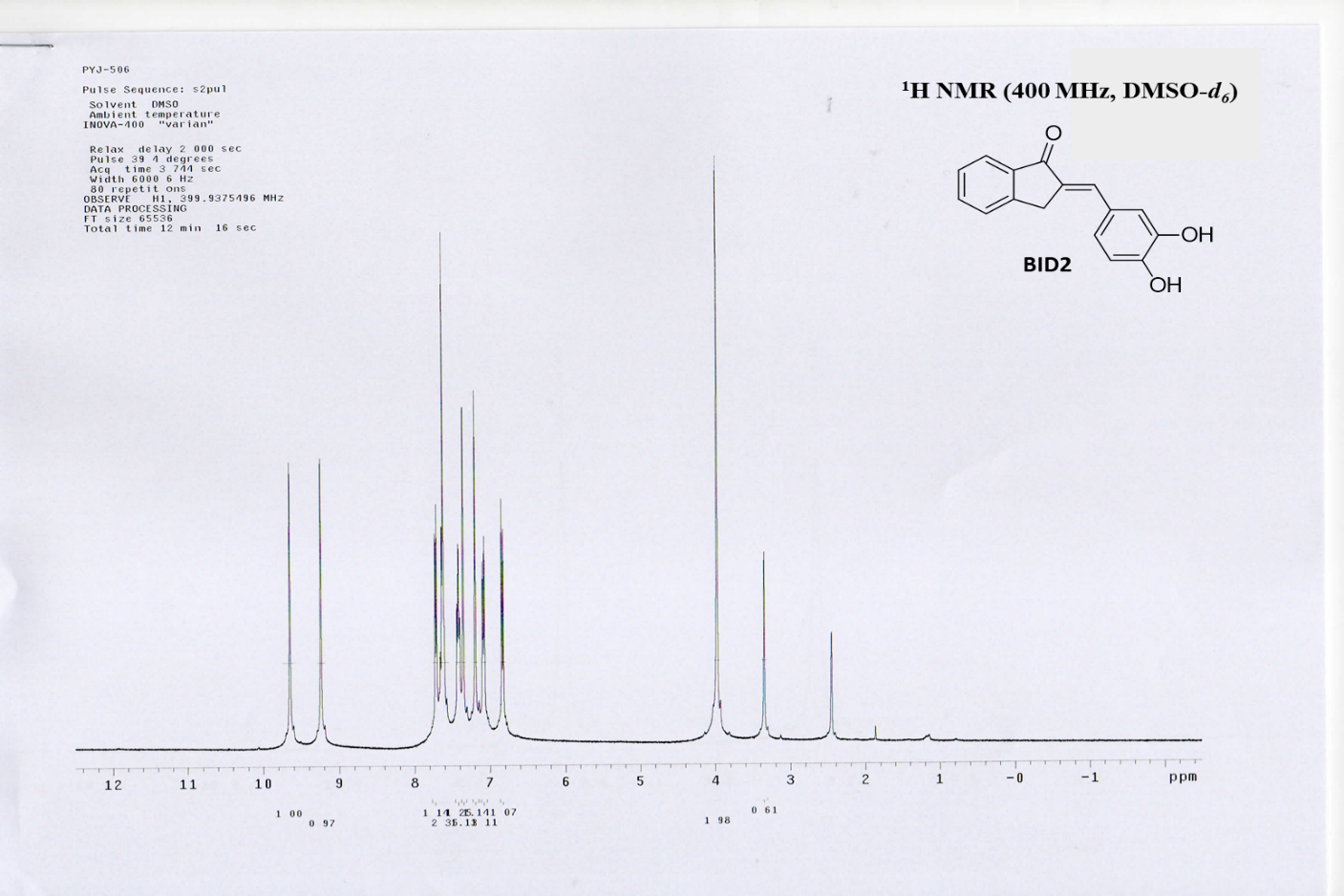


Fig. S4. ^1^H-NMR spectrum of **BID2**.


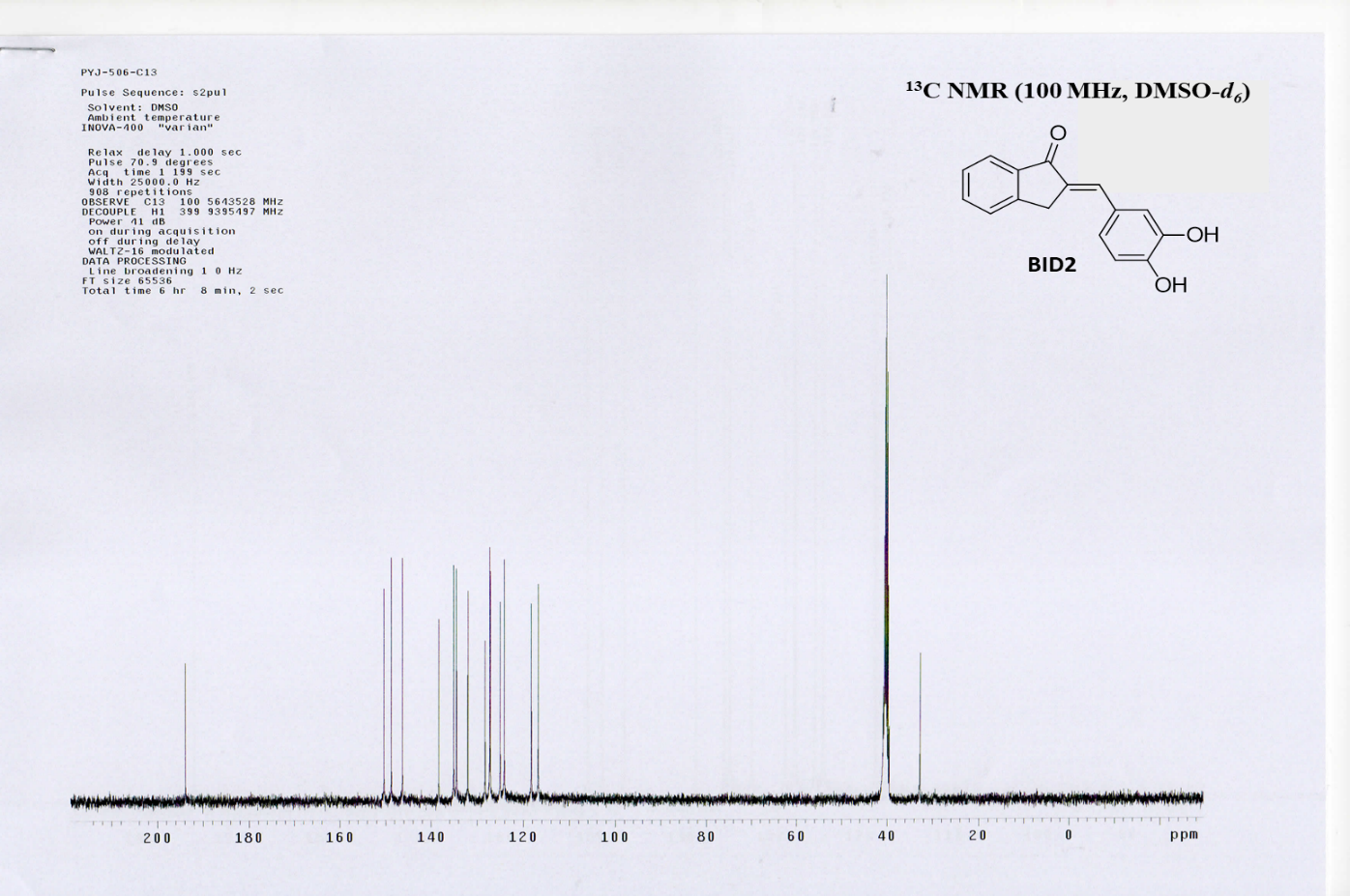


Fig. S5. ^13^C-NMR spectrum of **BID2**.


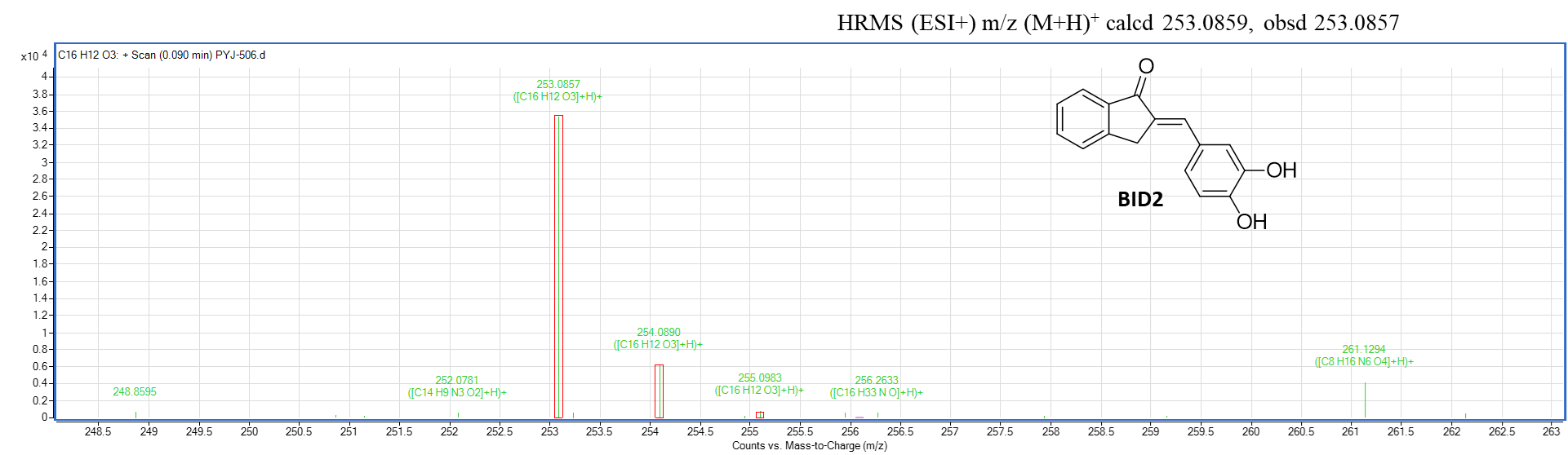


Fig. S6. ESI-MS spectrum of **BID2**.


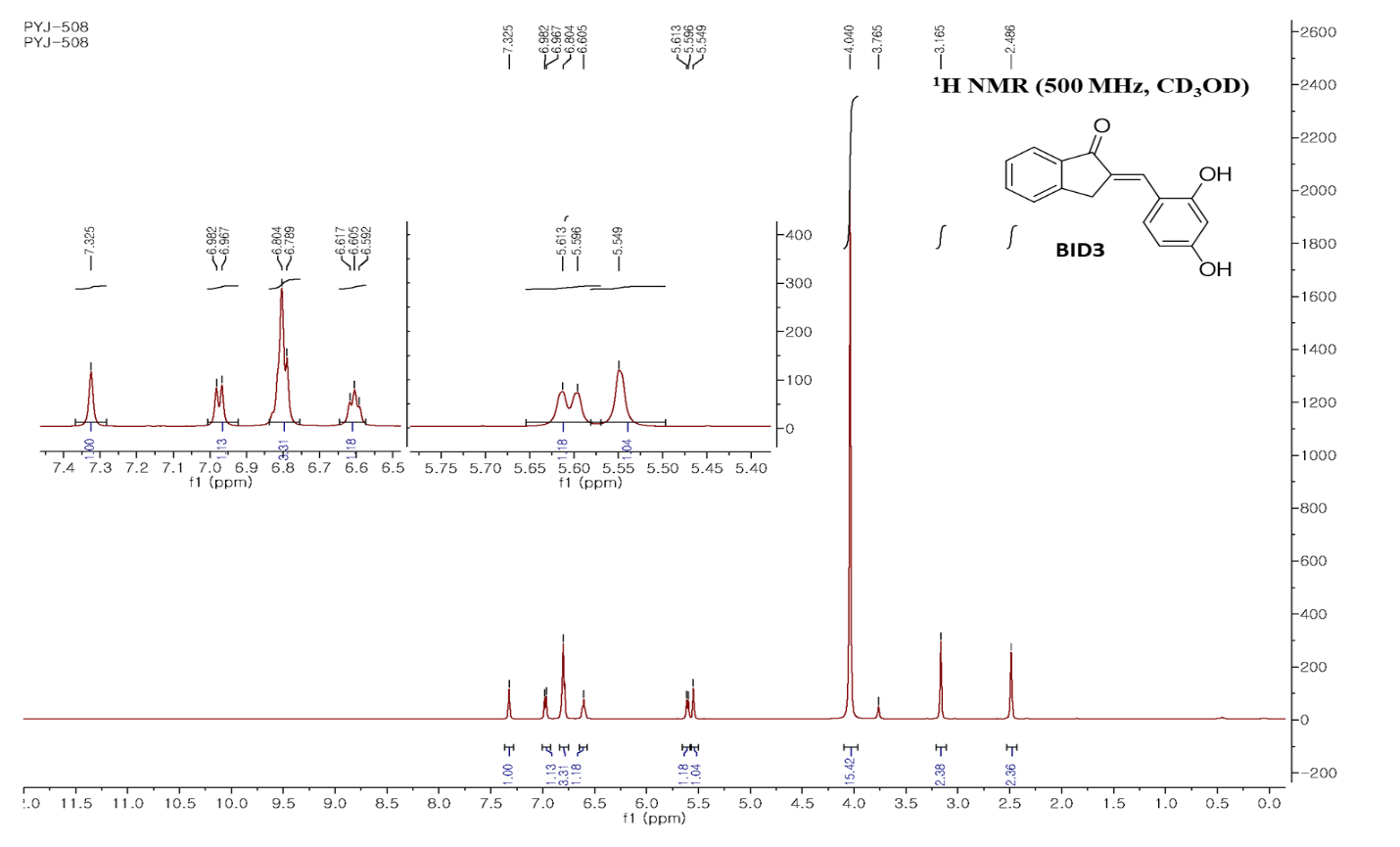


Fig. S7. ^1^H-NMR spectrum of **BID3**.


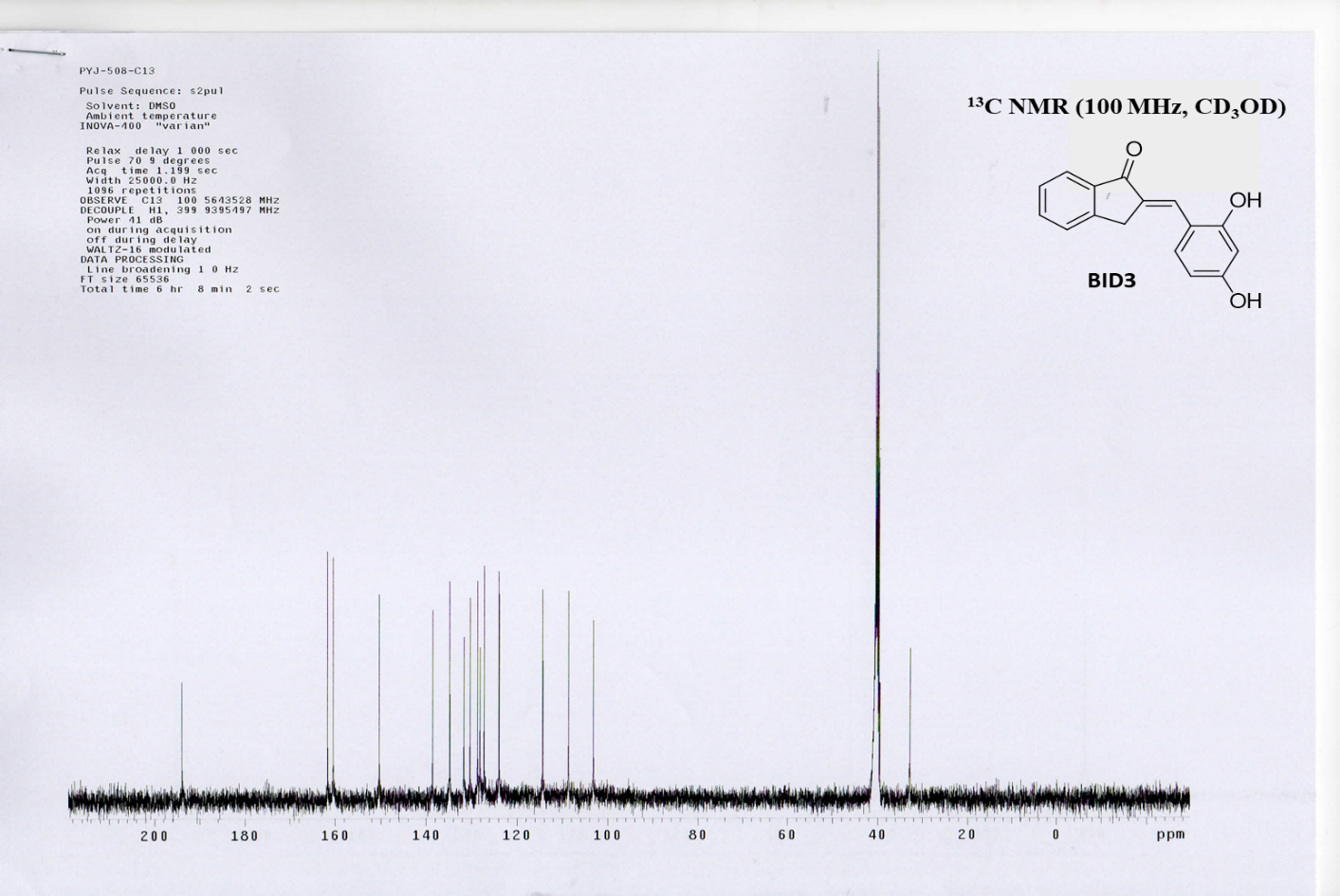


Fig. S8. ^13^C-NMR spectrum of **BID2**.


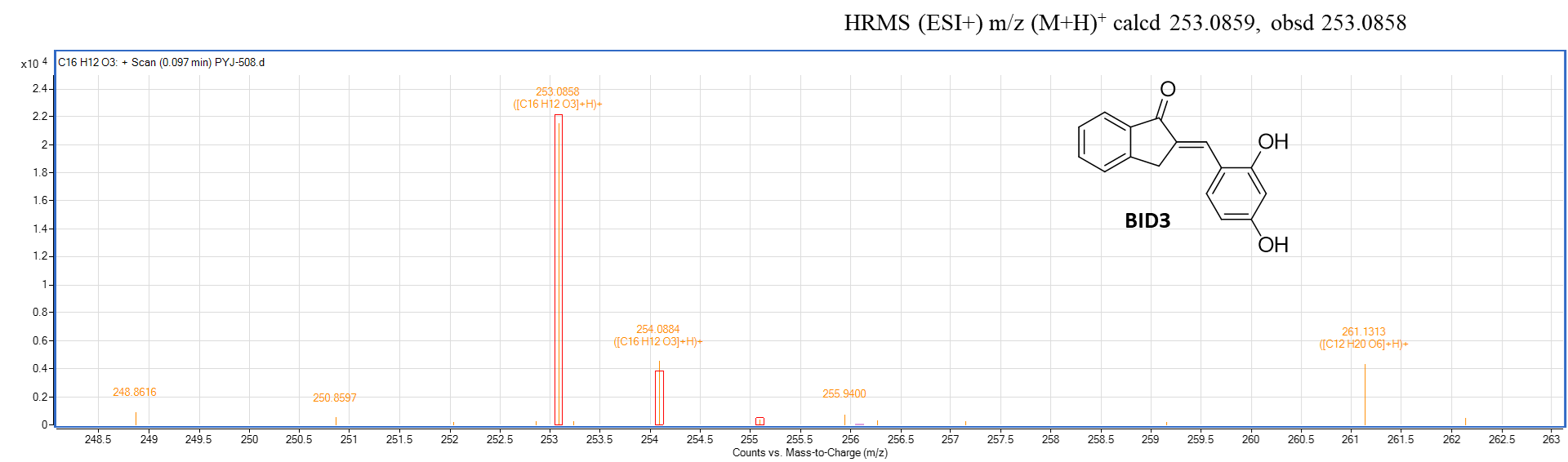


Fig. S9. ESI-MS spectrum of **BID3**.


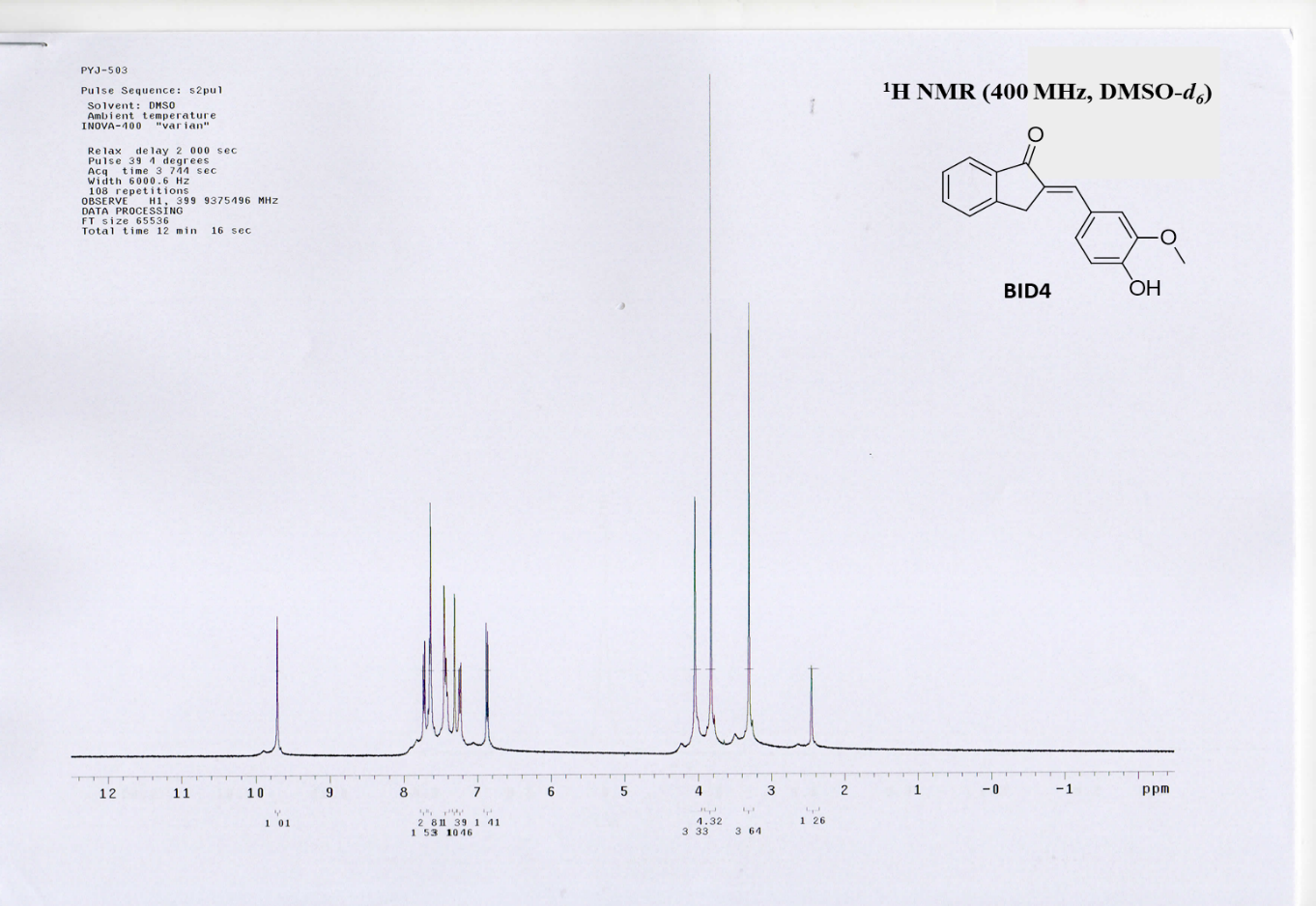


Fig. S10. ^1^H-NMR spectrum of **BID4**.


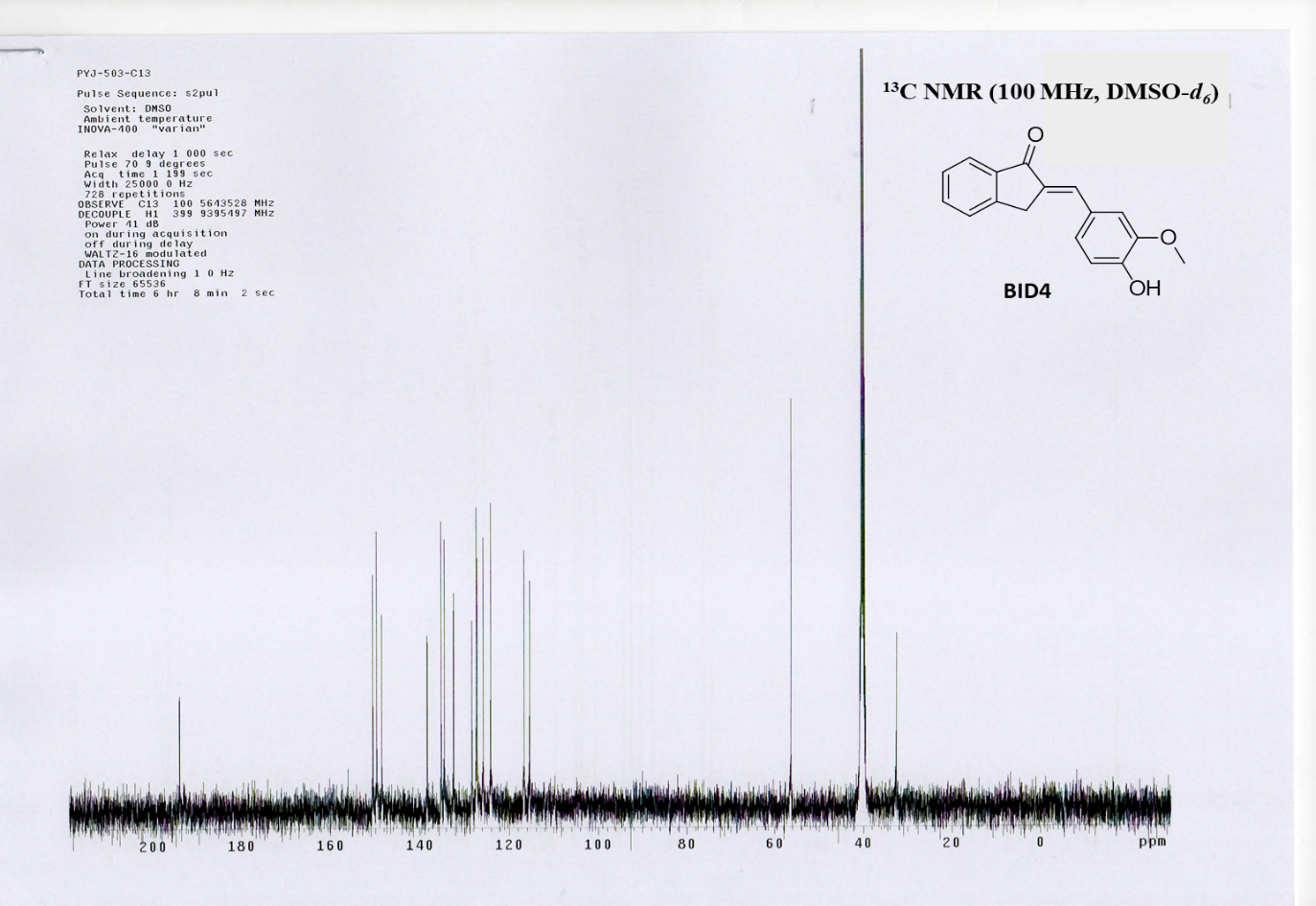


Fig. S11. ^13^C-NMR spectrum of **BID4**.


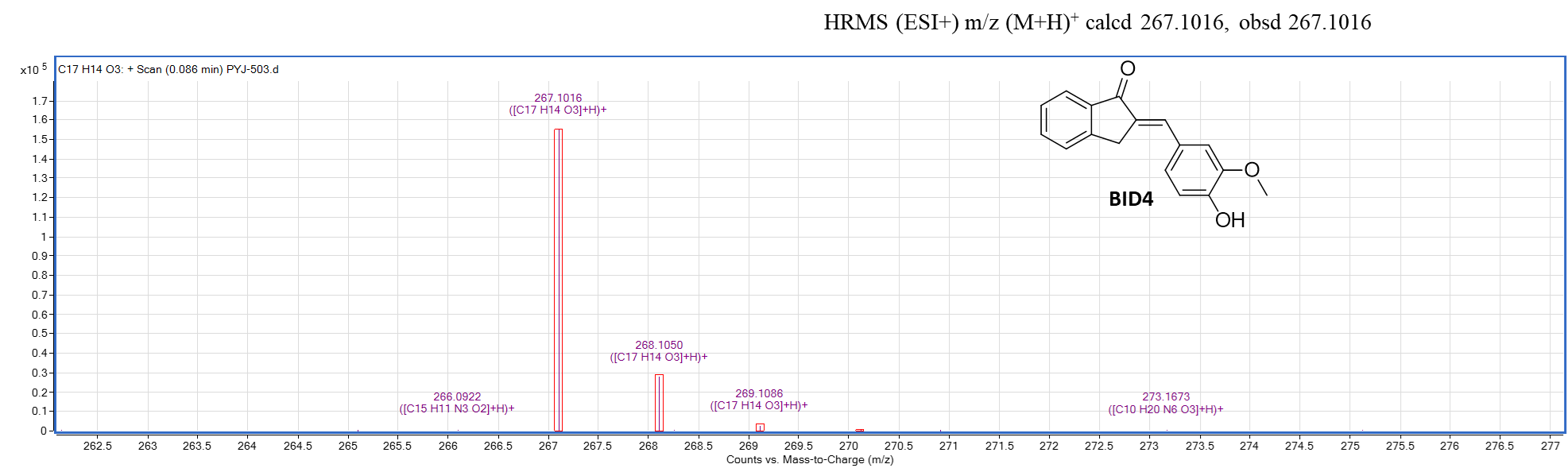


Fig. S12. ESI-MS spectrum of **BID4**.


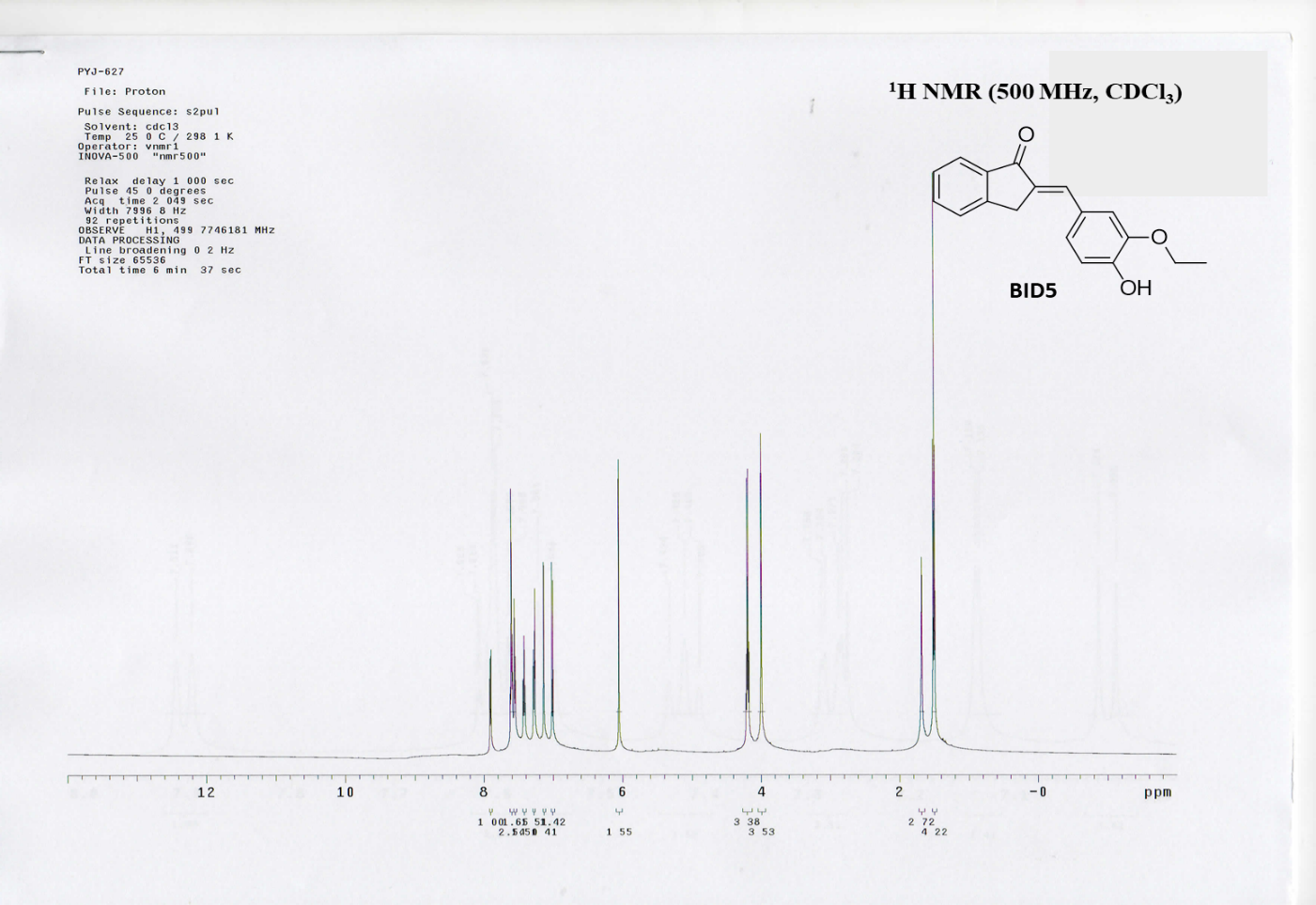


Fig. S13. ^1^H-NMR spectrum of **BID5**.


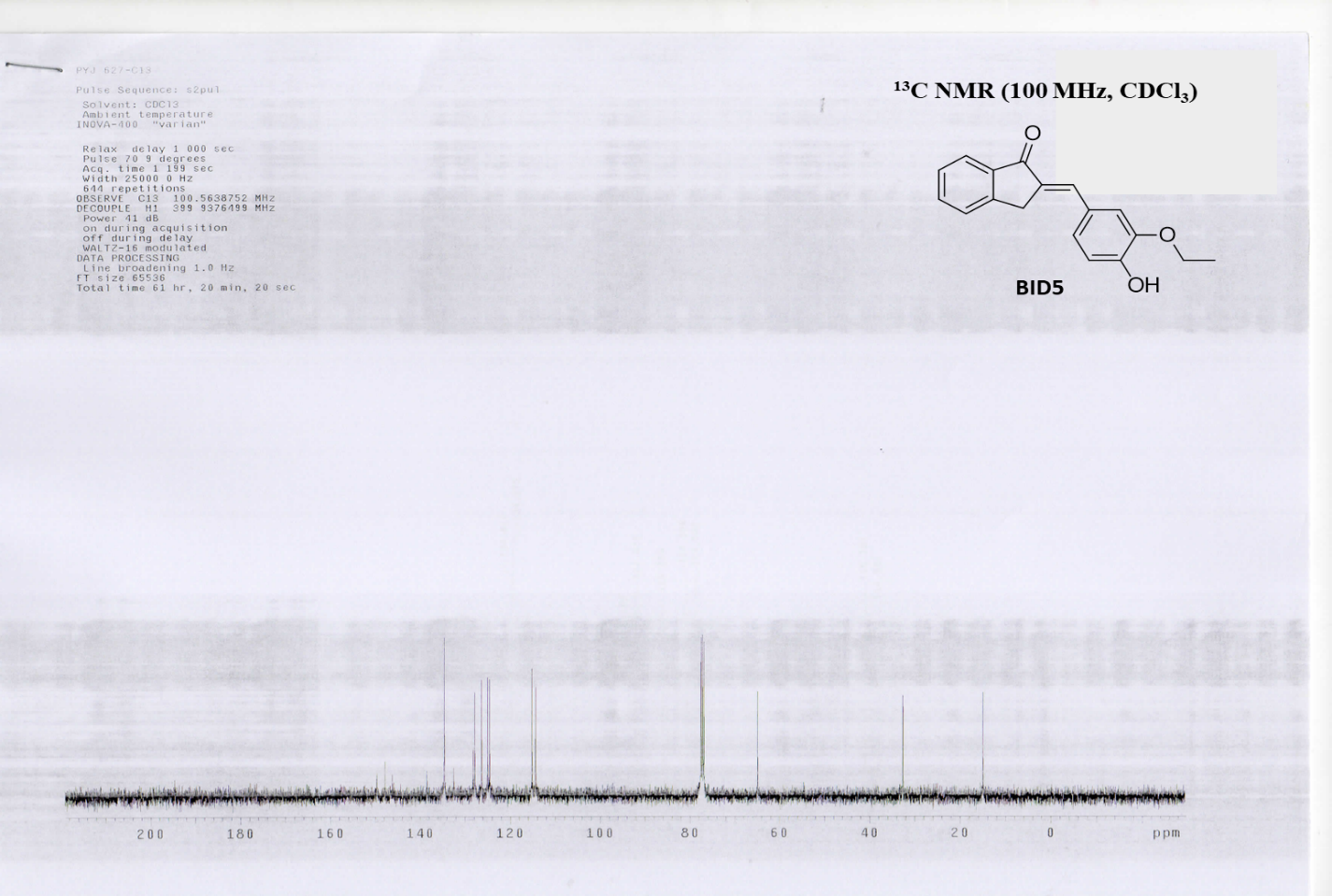


Fig. S14. ^13^C-NMR spectrum of **BID5**.


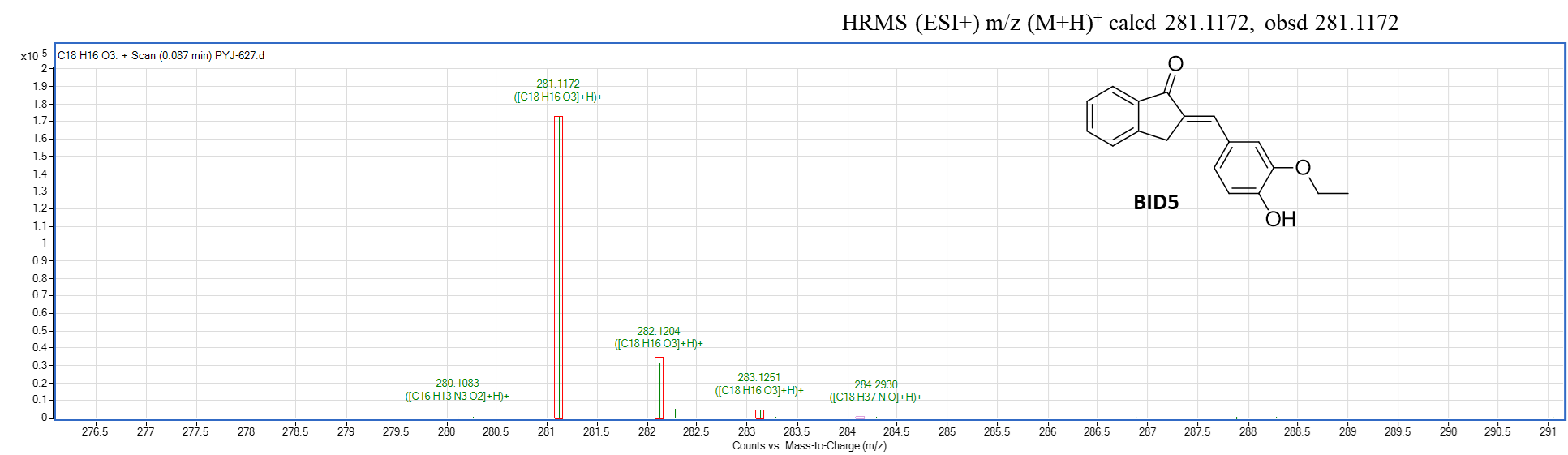


Fig. S15. ESI-MS spectrum of **BID5**.

Fig. S16. ^1^H-NMR spectrum of **BID6**.
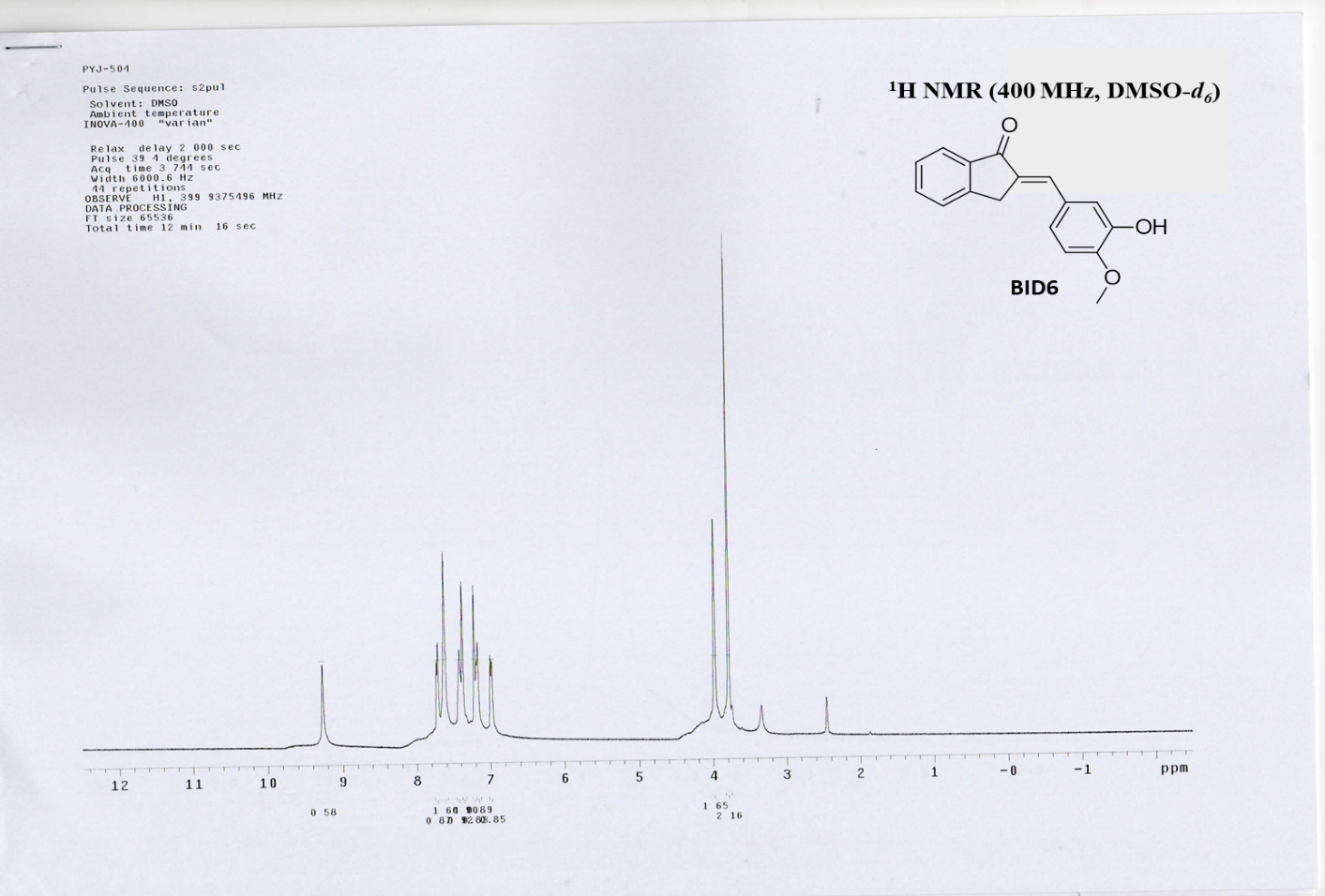


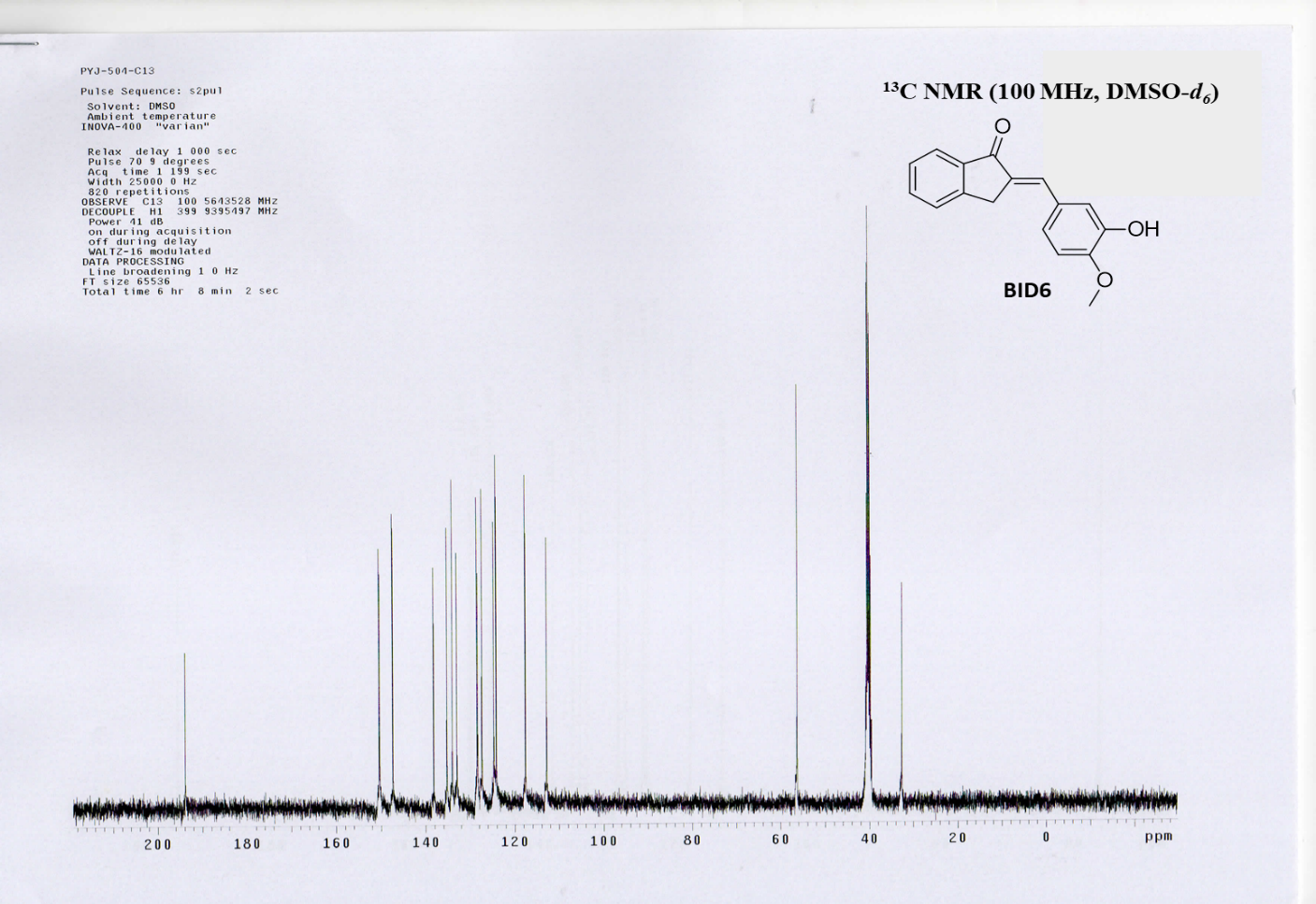


Fig. S17. ^13^C-NMR spectrum of **BID6**.


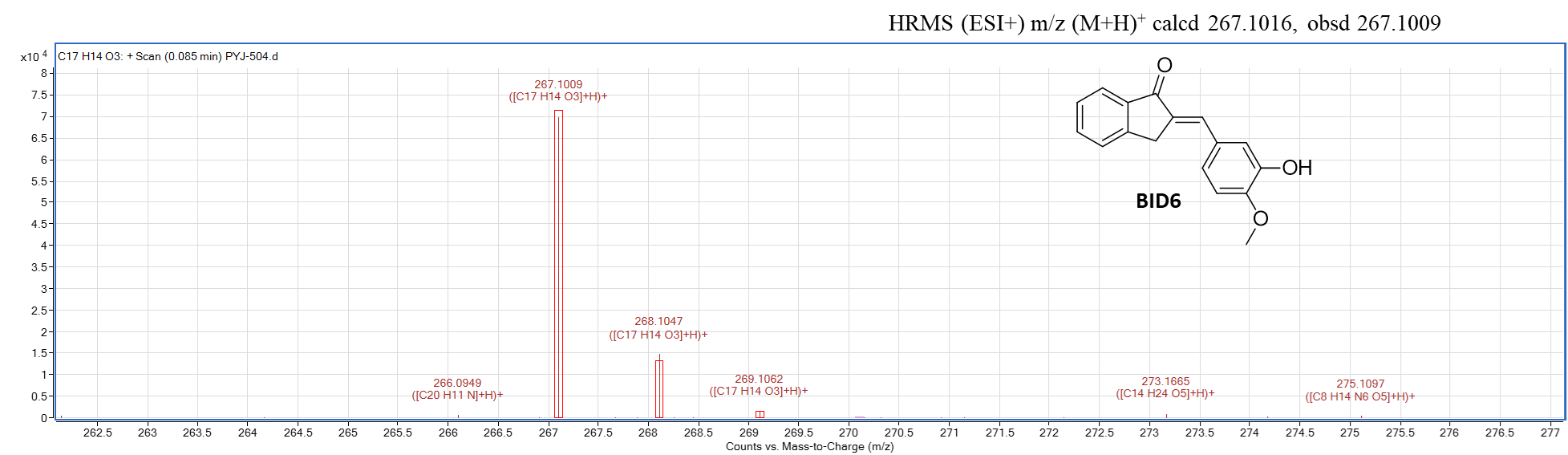


Fig. S18. ESI-MS spectrum of **BID6**.


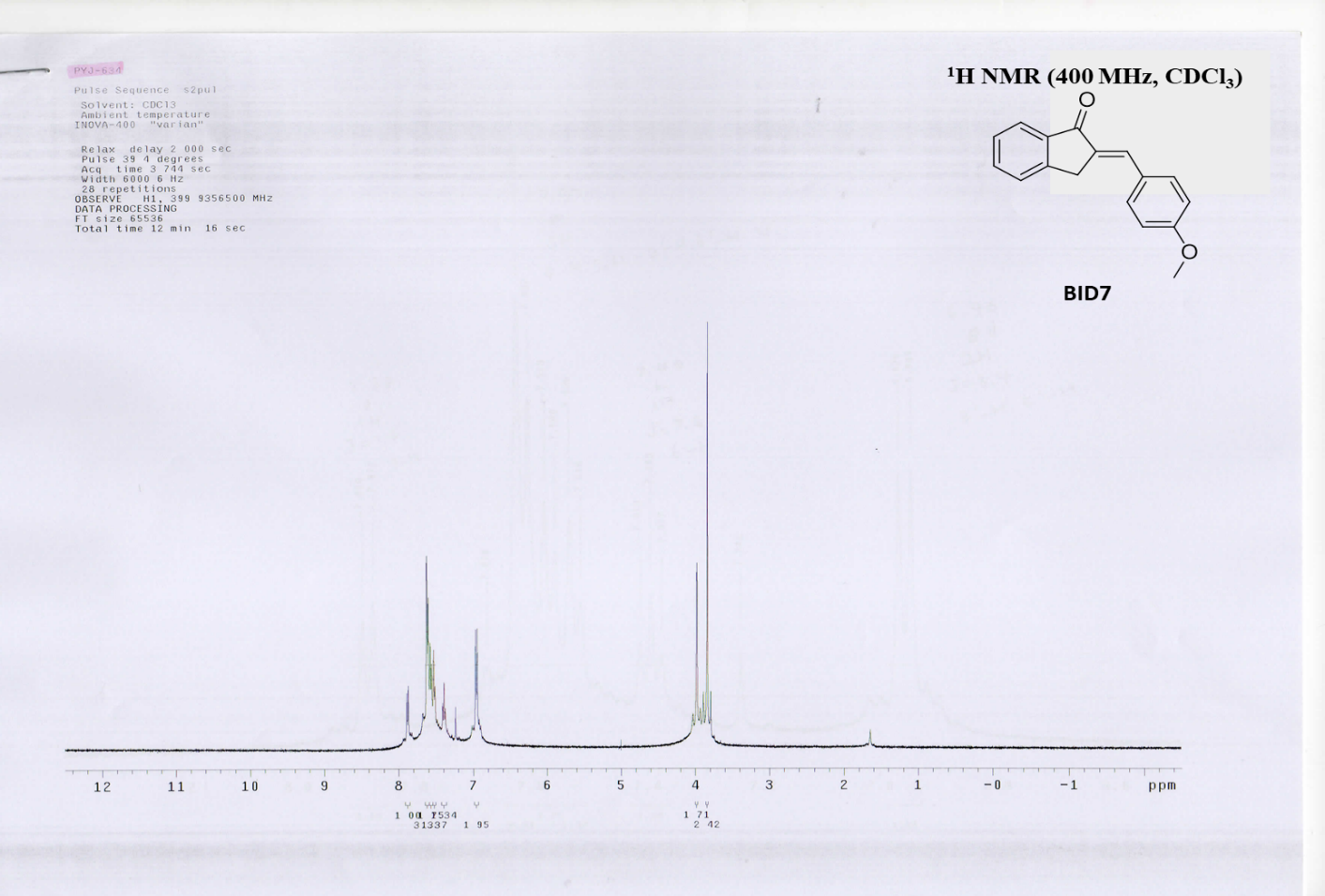


Fig. S19. ^1^H-NMR spectrum of **BID7**.


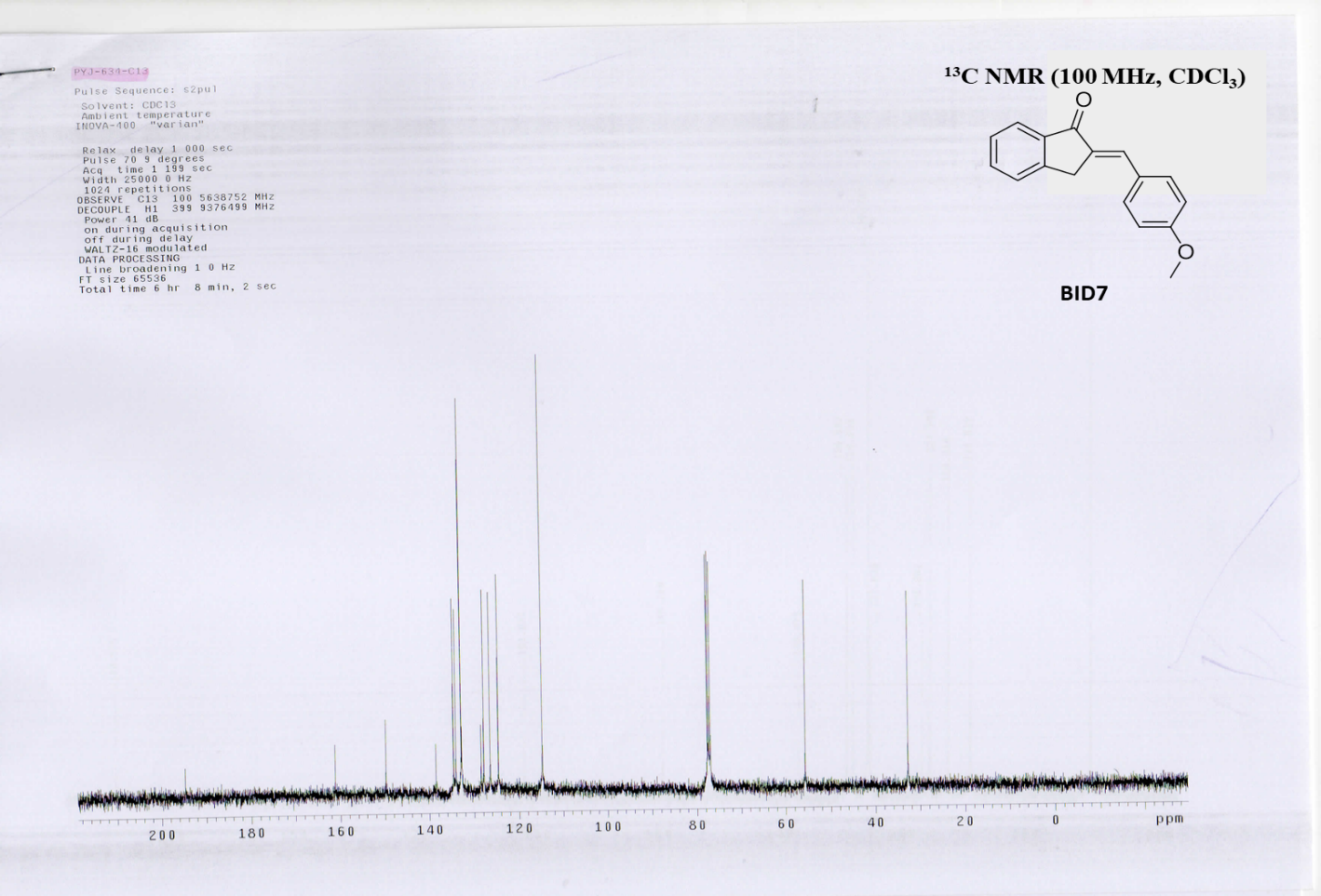


Fig. S20. ^13^C-NMR spectrum of **BID7**


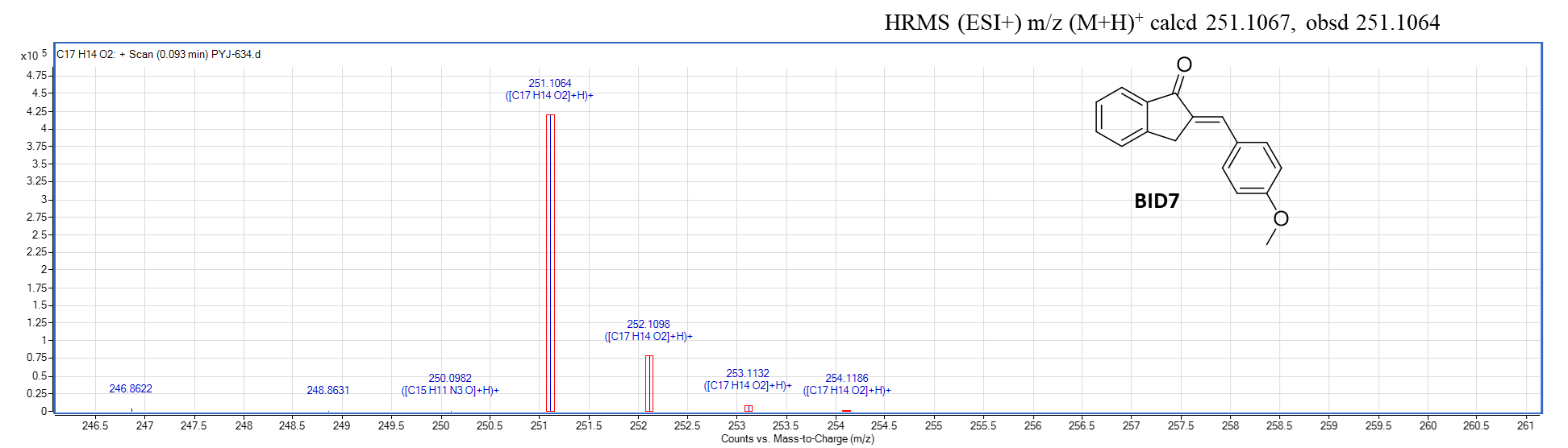


Fig. S21. ESI-MS spectrum of **BID7**.


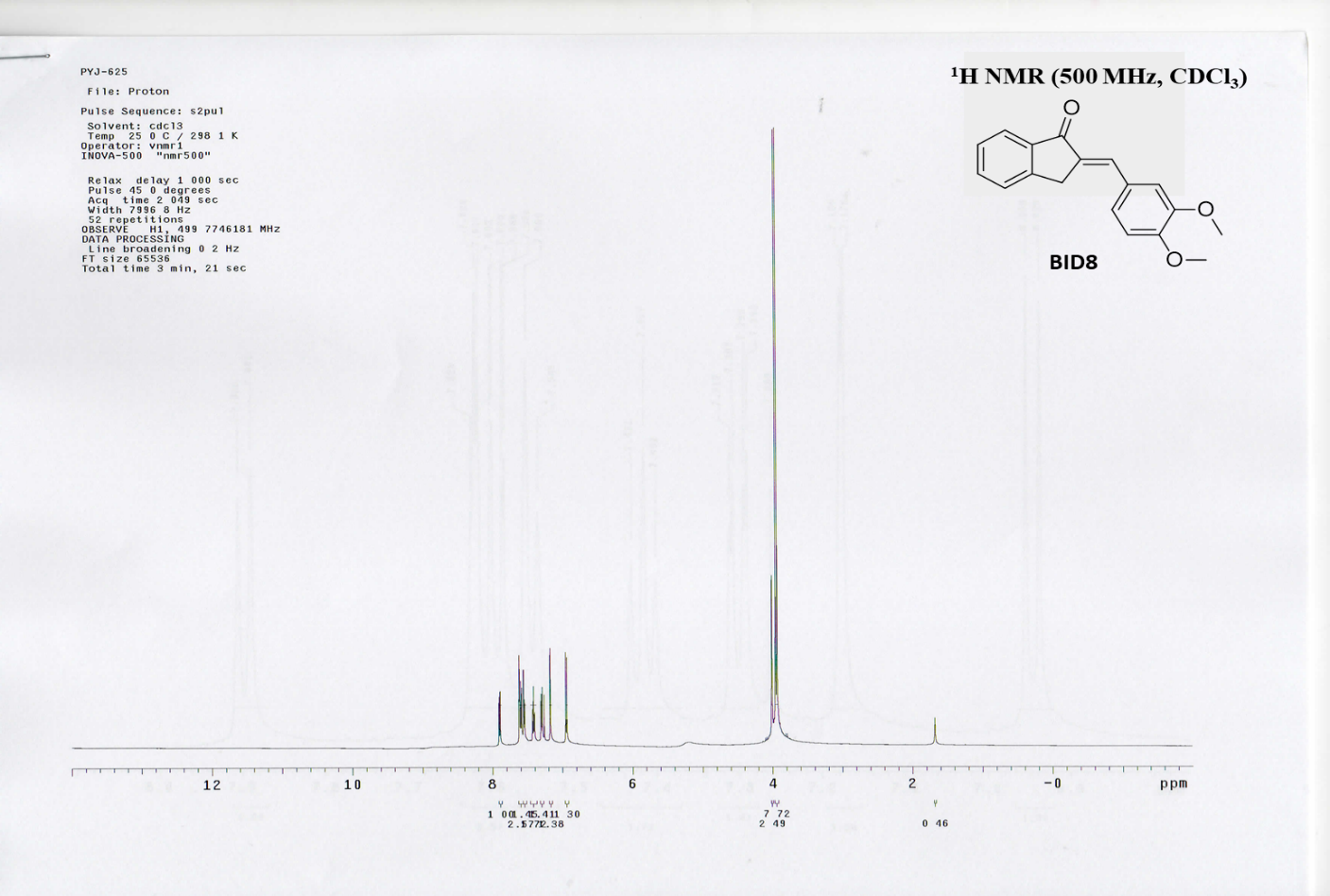


Fig. S22. ^1^H-NMR spectrum of **BID8**.


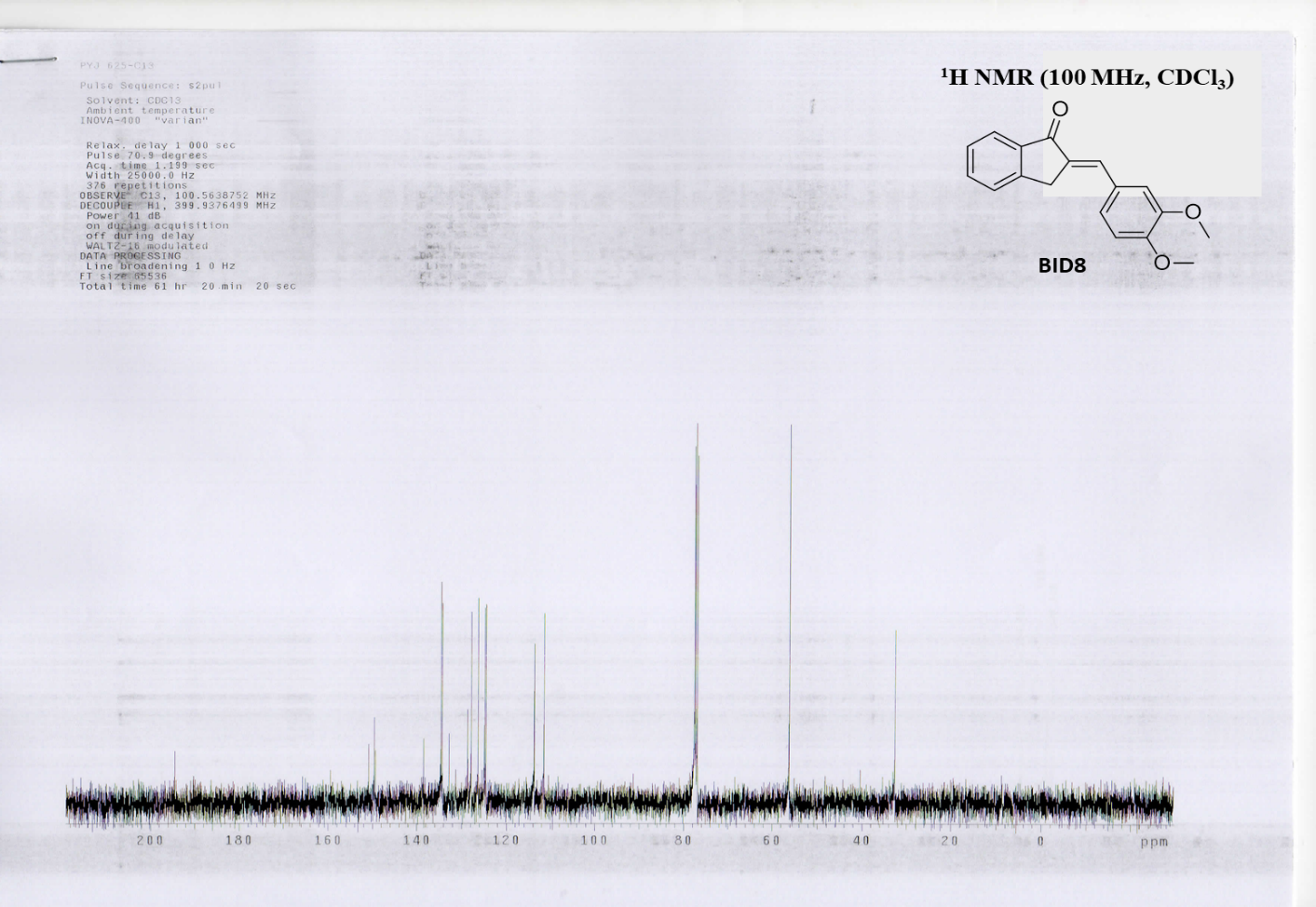


Fig. S23. ^13^C-NMR spectrum of **BID8**


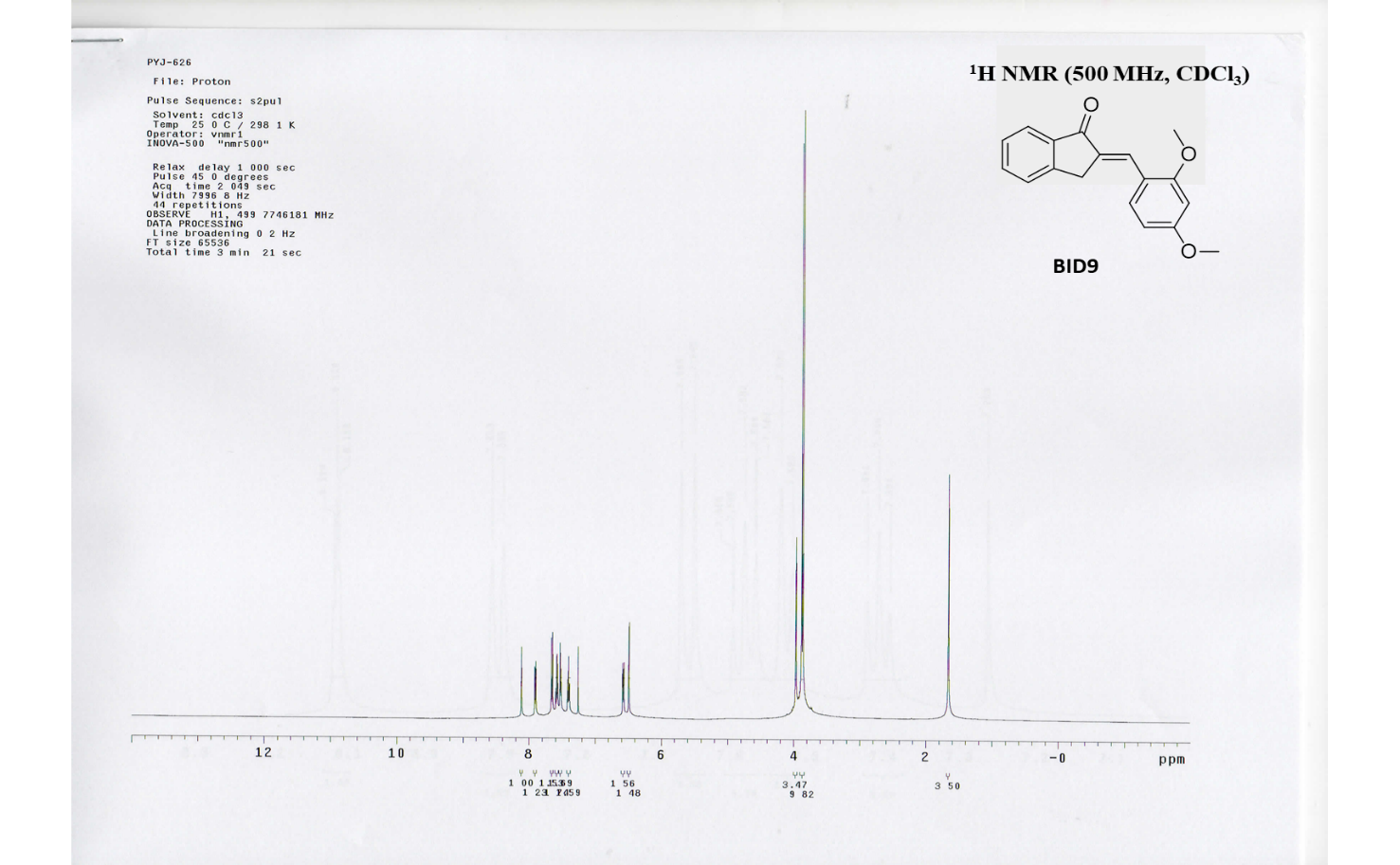


Fig. S24. ^1^H-NMR spectrum of **BID9**.


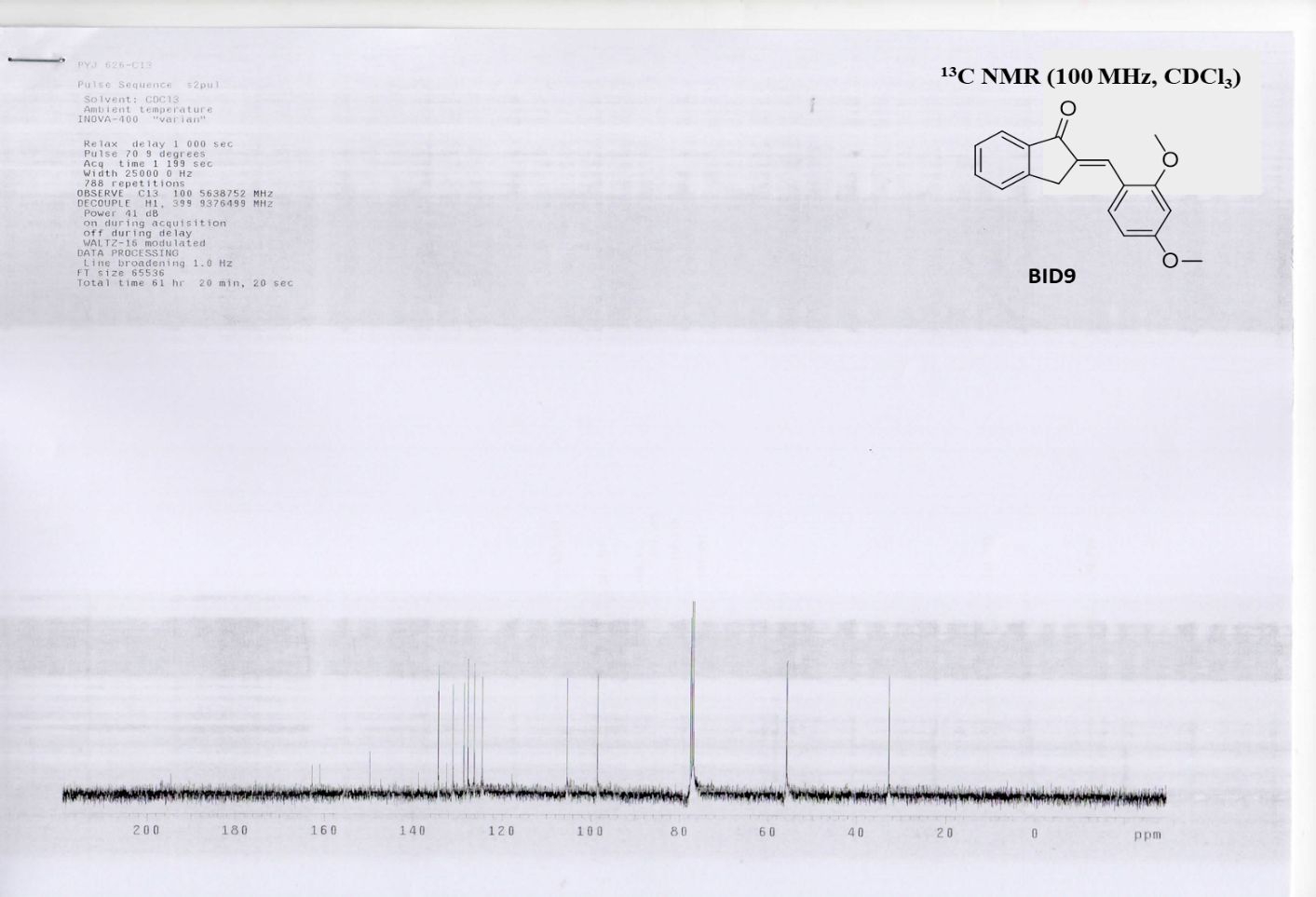


Fig. S25. ^13^C-NMR spectrum of **BID9**


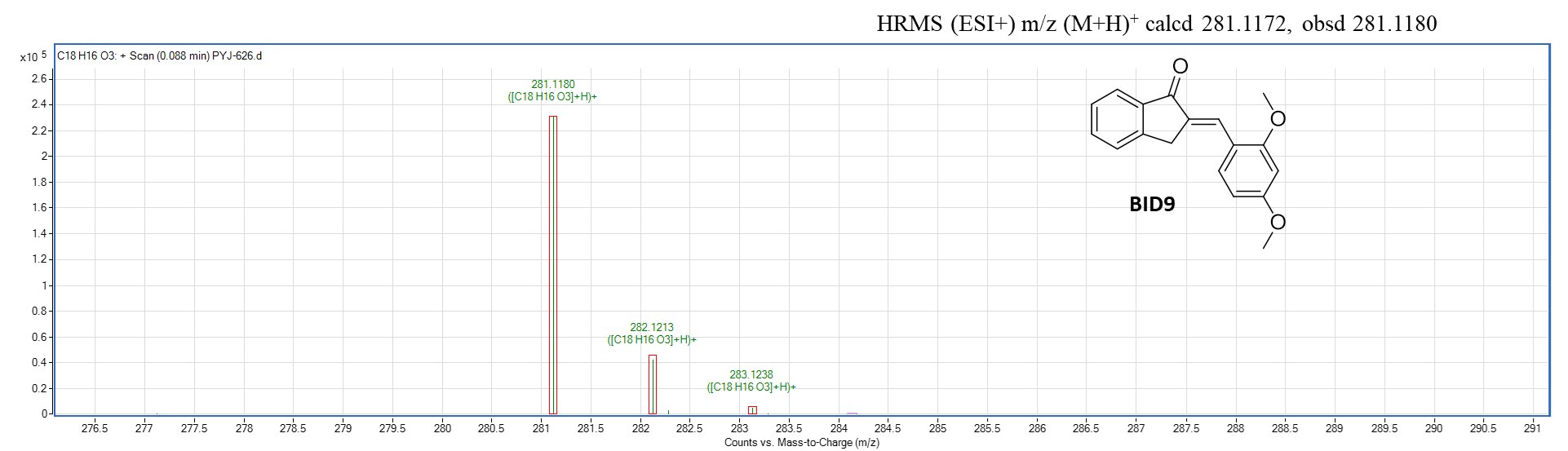


Fig. S26. ESI-MS spectrum of **BID9**.


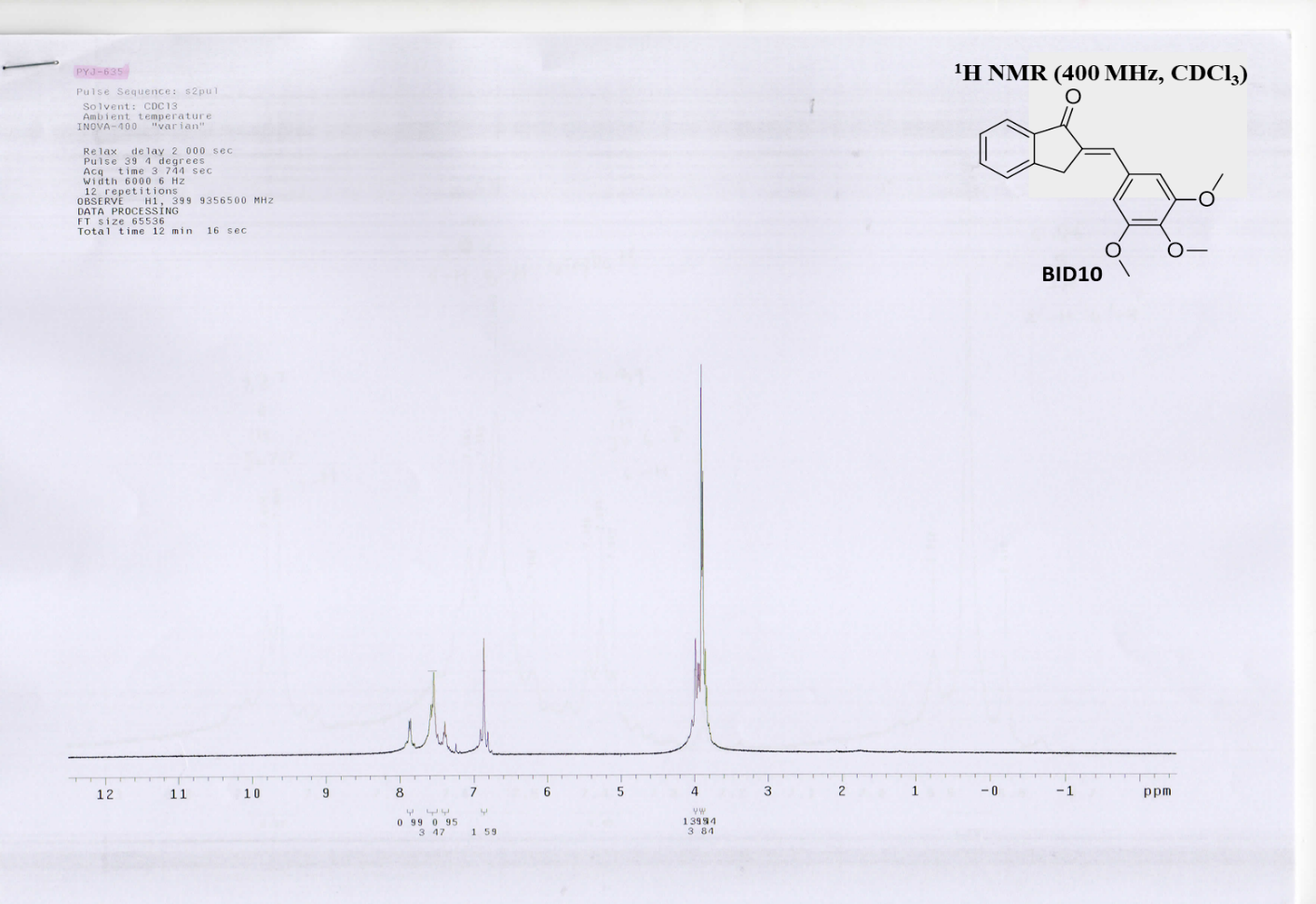


Fig. S27. ^1^H-NMR spectrum of **BID10**.


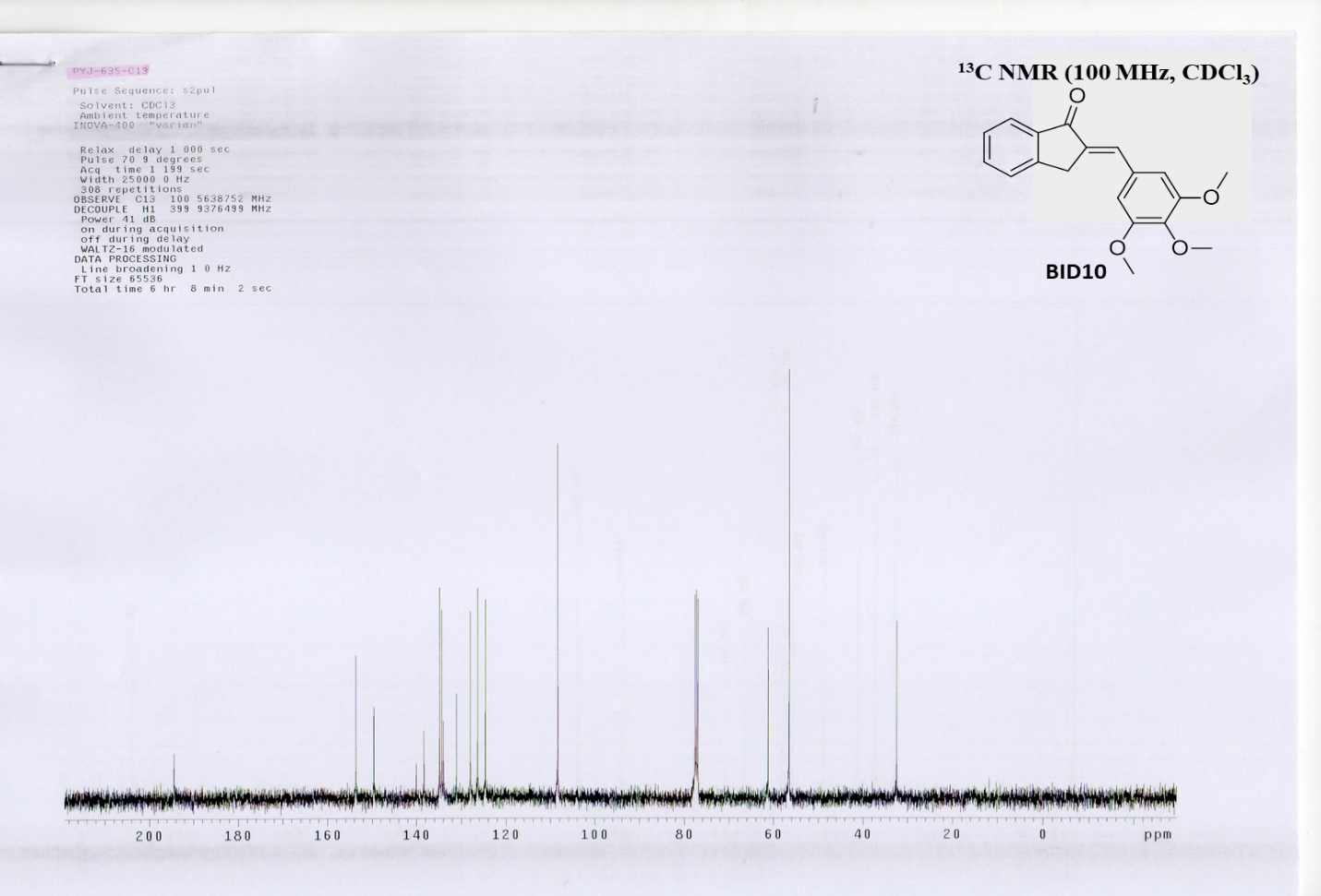


Fig. S28. ^13^C-NMR spectrum of **BID10**.


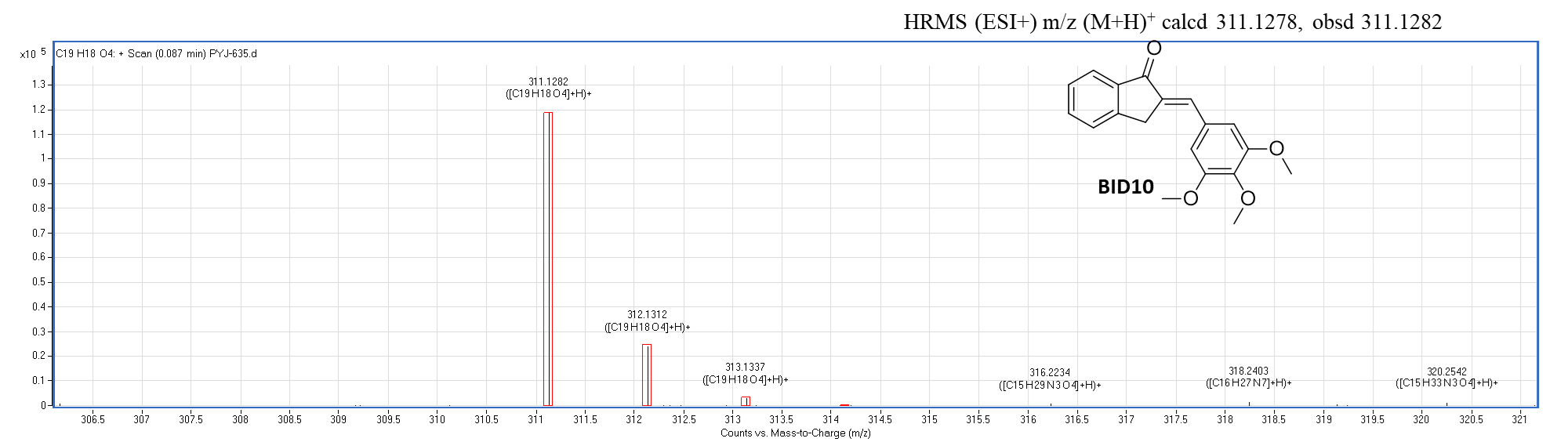


Fig. S29. ESI-MS spectrum of **BID10**.


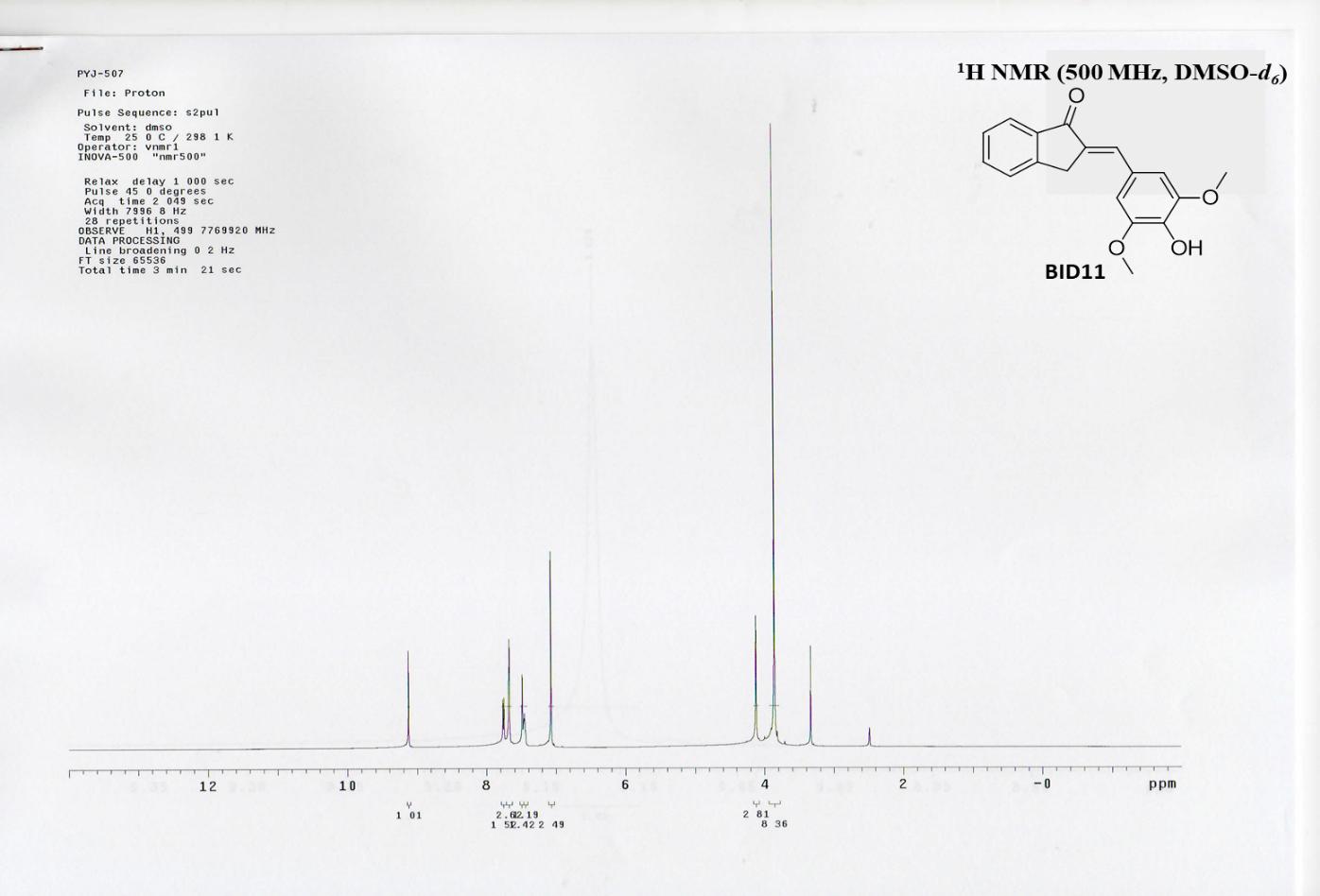


Fig. S30. ^1^H-NMR spectrum of **BID11**.


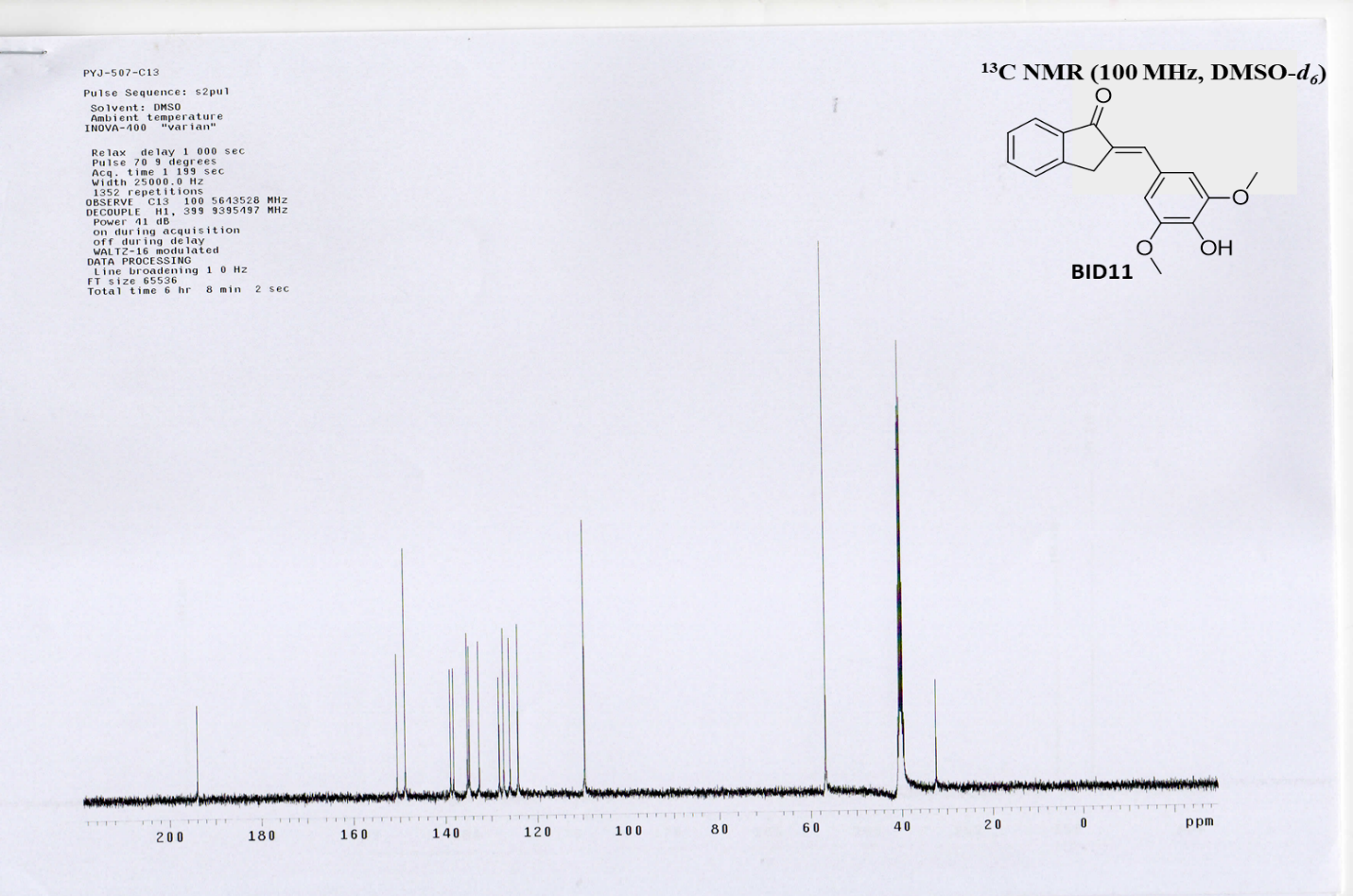


Fig. S31. ^13^C-NMR spectrum of **BID11**.


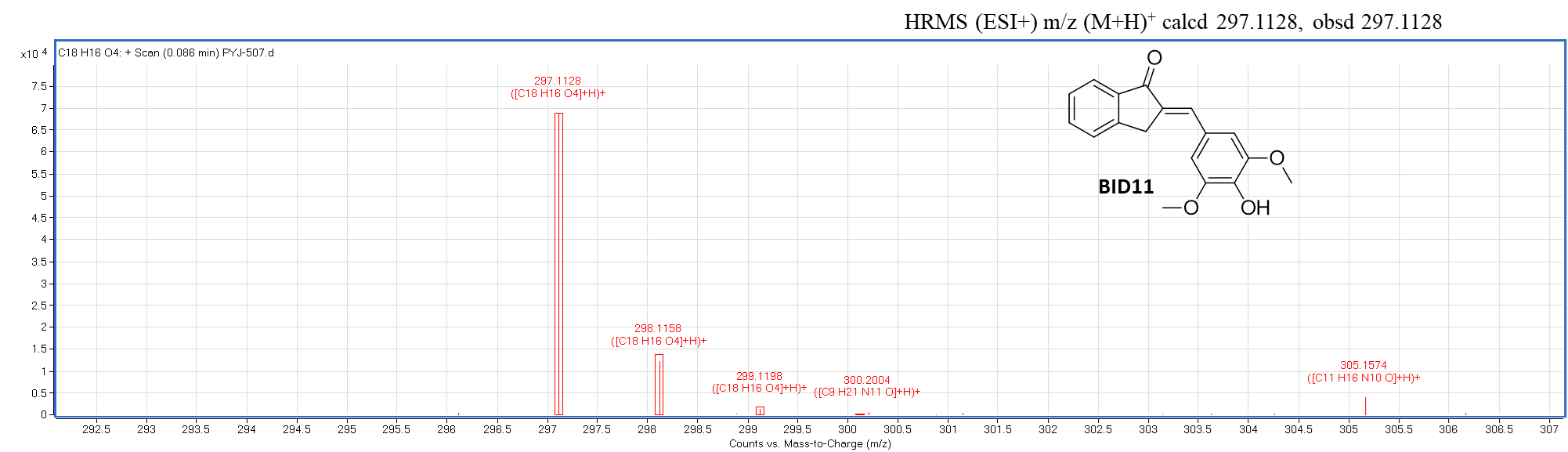


Fig. S32. ESI-MS spectra of **BID11**.

**
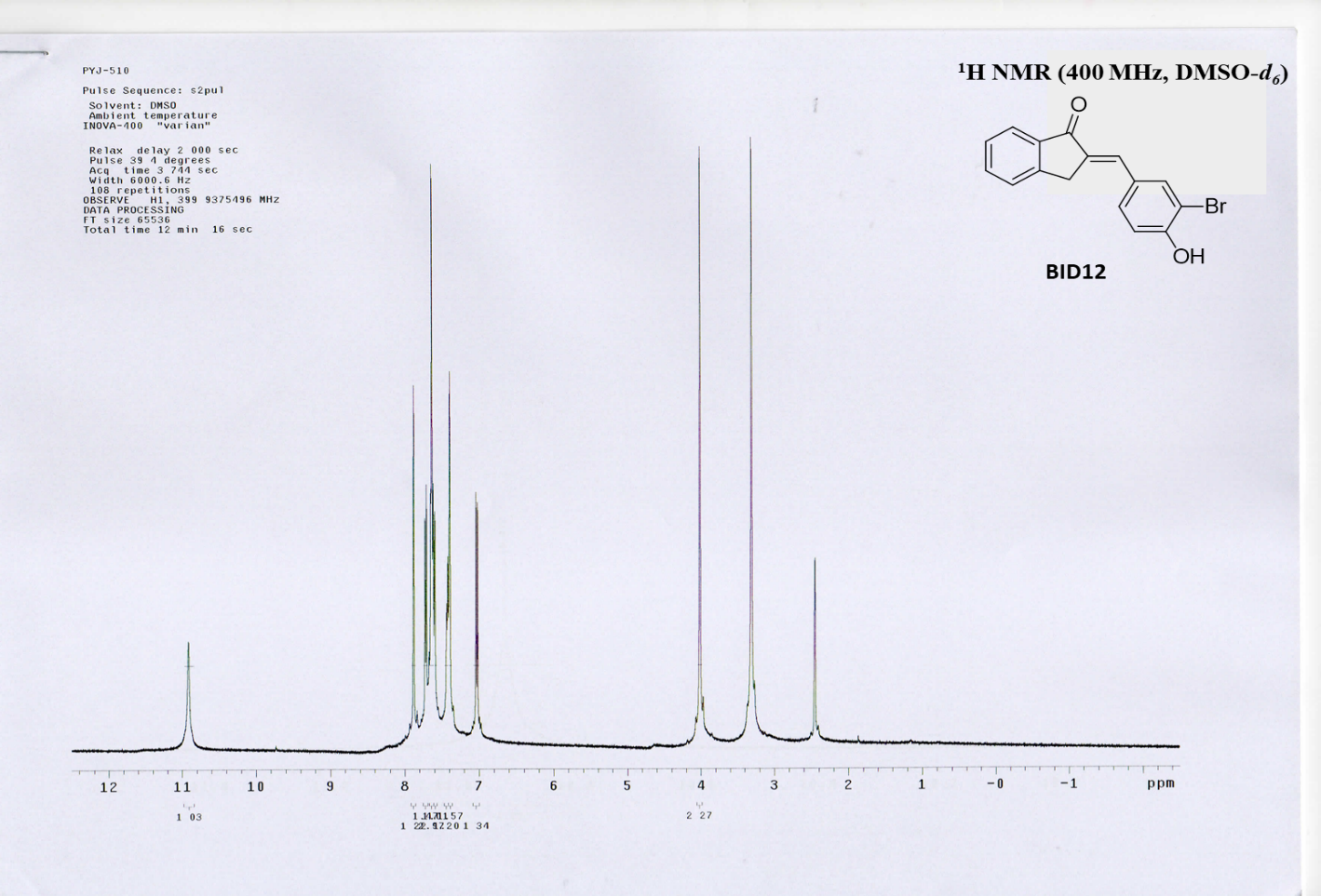
**

Fig. S33. ^1^H-NMR spectrum of **BID12**.


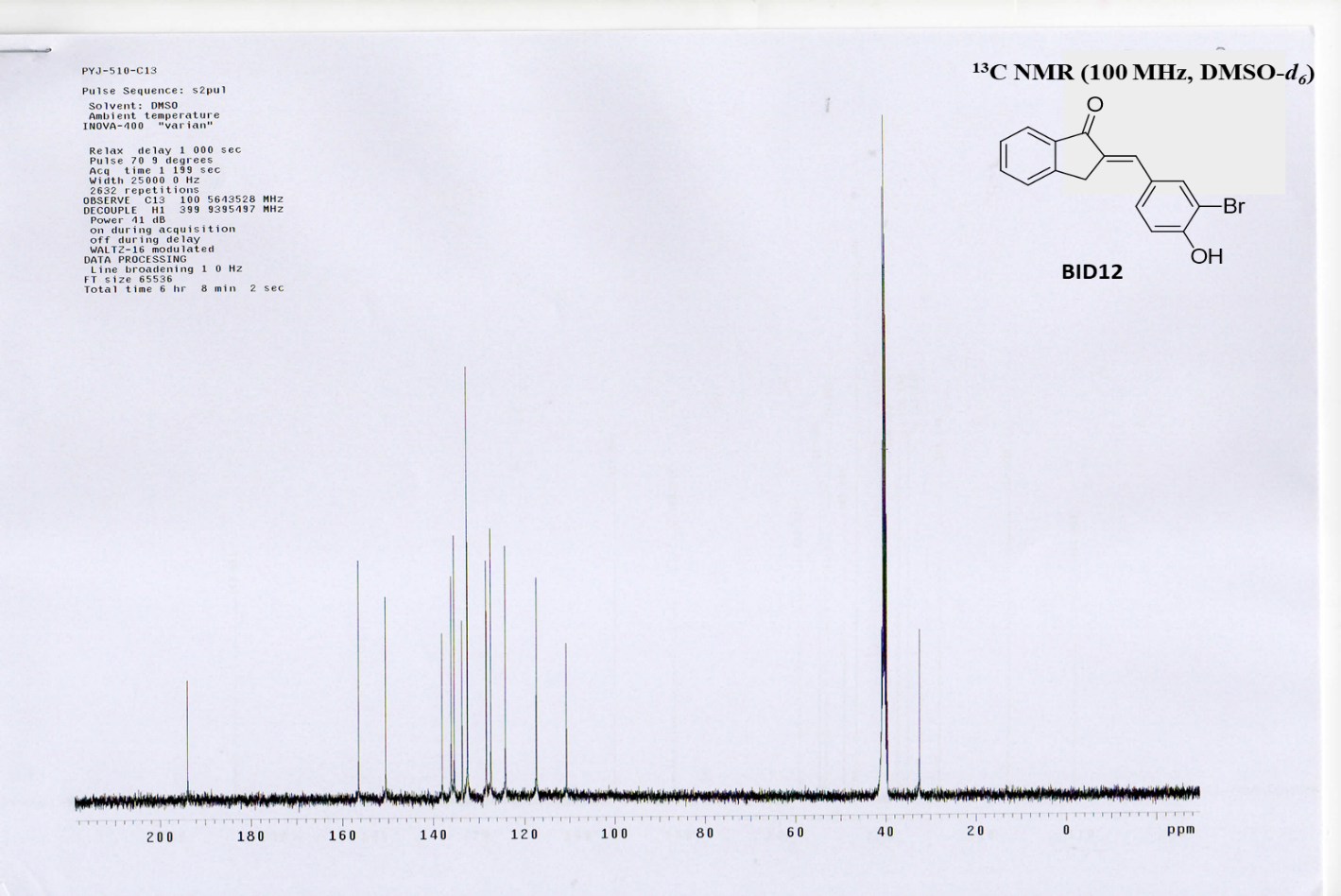


Fig. S34. ^13^C-NMR spectrum of **BID12**.


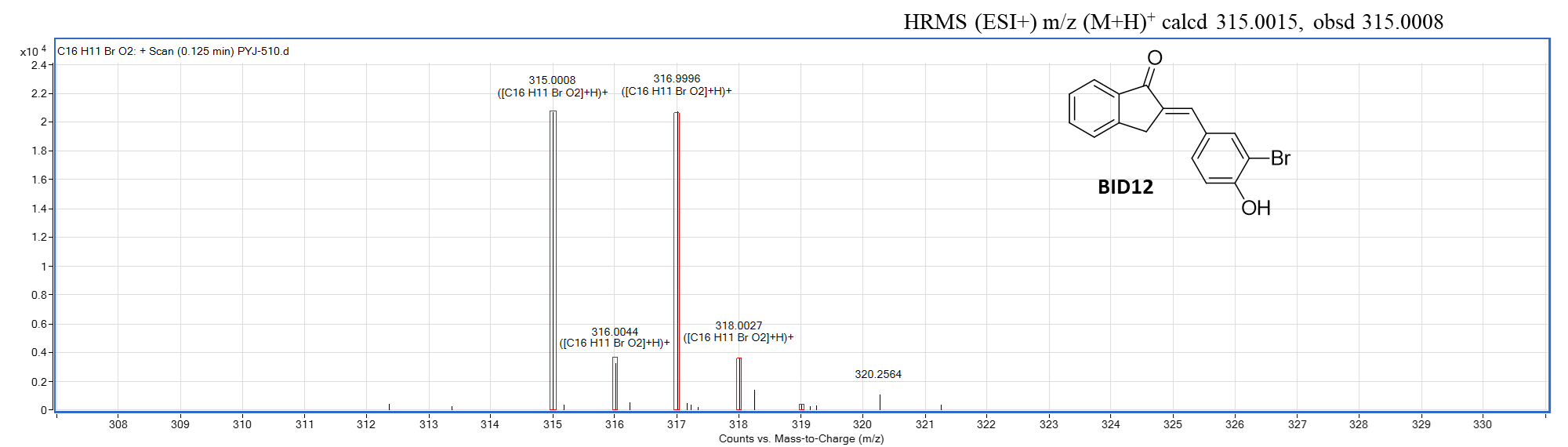


Fig. S35. ESI-MS spectra of **BID12**.


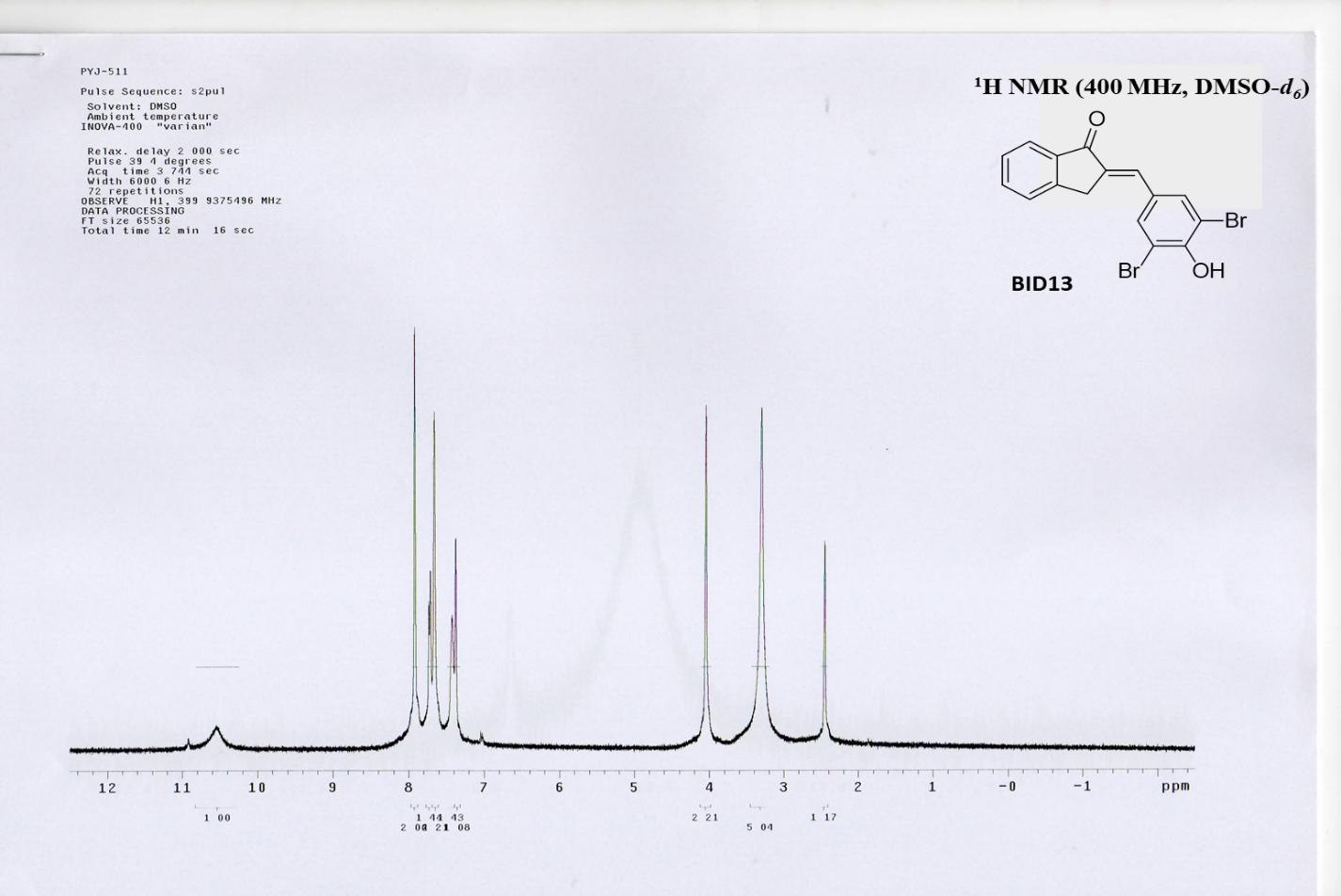


Fig. S36. ^1^H-NMR spectrum of **BID13**.


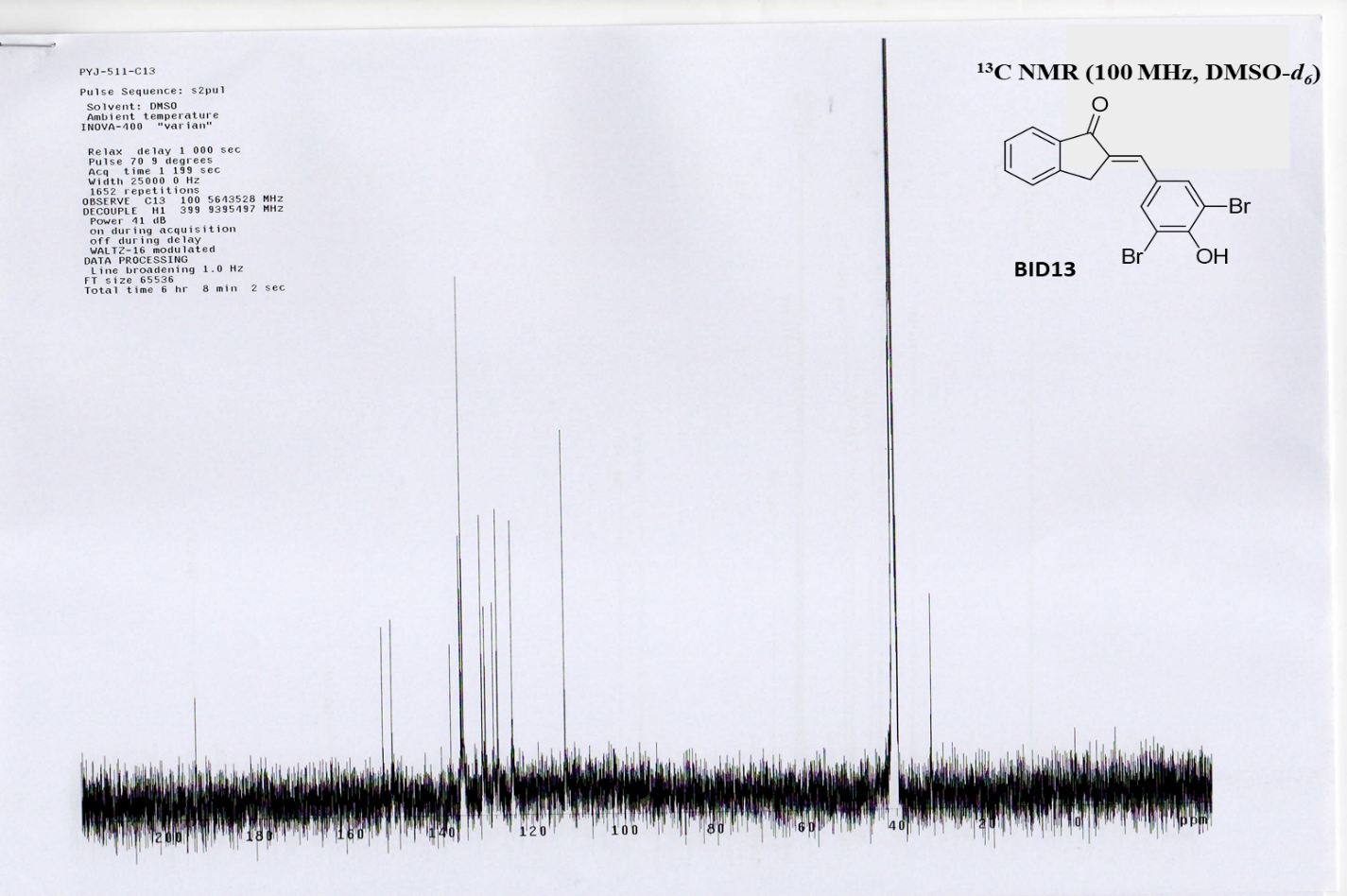


Fig. S37. ^13^C-NMR spectrum of **BID13**.


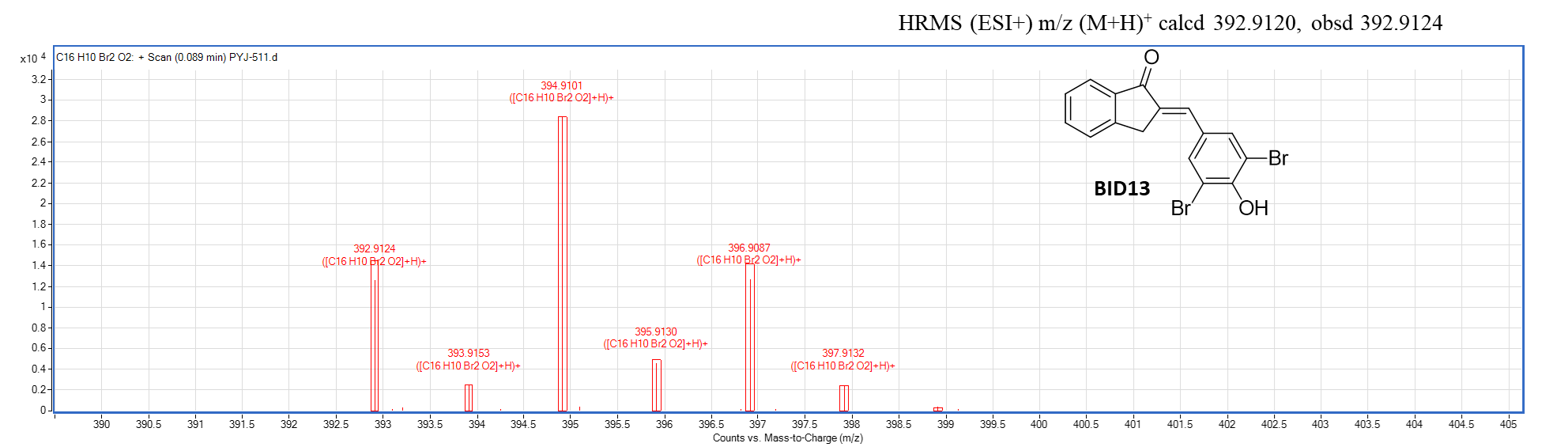


Fig. S38. ESI-MS spectra of **BID13**.
